# Extensive expansion and diversification of the chemokine gene family in zebrafish: Identification of a novel chemokine subfamily CX

**DOI:** 10.1186/1471-2164-9-222

**Published:** 2008-05-15

**Authors:** Hisayuki Nomiyama, Kunio Hieshima, Naoki Osada, Yoko Kato-Unoki, Kaori Otsuka-Ono, Sumio Takegawa, Toshiaki Izawa, Akio Yoshizawa, Yutaka Kikuchi, Sumio Tanase, Retsu Miura, Jun Kusuda, Miki Nakao, Osamu Yoshie

**Affiliations:** 1 Department of Molecular Enzymology, Kumamoto University Graduate School of Medical Sciences, Kumamoto 860-8556, Japan; 2 Department of Microbiology, Kinki University School of Medicine, Osaka-Sayama, Osaka 589-8511, Japan; 3 Department of Biomedical Resources, National Institute of Biomedical Innovation, Ibaraki, Osaka 567-0085, Japan; 4 Laboratory of Marine Biochemistry, Department of Bioscience and Biotechnology, Graduate School of Bioresource and Bioenvironmental Science, Kyushu University, Fukuoka 812-8581, Japan; 5 Division of Biological Science, Graduate School of Science, Nagoya University, Nagoya 464-8602, Japan; 6 Department of Analytical Biochemistry, Kumamoto University School of Health Sciences, Kumamoto 862-0976, Japan; 7 Department of Biological Science, Graduate School of Science, Hiroshima University, Higashi-Hiroshima 739-8526, Japan

## Abstract

**Background:**

The chemokine family plays important roles in cell migration and activation. In humans, at least 44 members are known. Based on the arrangement of the four conserved cysteine residues, chemokines are now classified into four subfamilies, CXC, CC, XC and CX3C. Given that zebrafish is an important experimental model and teleost fishes constitute an evolutionarily diverse group that forms half the vertebrate species, it would be useful to compare the zebrafish chemokine system with those of mammals. Prior to this study, however, only incomplete lists of the zebrafish chemokine genes were reported.

**Results:**

We systematically searched chemokine genes in the zebrafish genome and EST databases, and identified more than 100 chemokine genes. These genes were CXC, CC and XC subfamily members, while no CX3C gene was identified. We also searched chemokine genes in pufferfish fugu and *Tetraodon*, and found only 18 chemokine genes in each species. The majority of the identified chemokine genes are unique to zebrafish or teleost fishes. However, several groups of chemokines are moderately similar to human chemokines, and some chemokines are orthologous to human homeostatic chemokines CXCL12 and CXCL14. Zebrafish also possesses a novel species-specific subfamily consisting of five members, which we term the CX subfamily. The CX chemokines lack one of the two N-terminus conserved cysteine residues but retain the third and the fourth ones. (Note that the XC subfamily only retains the second and fourth of the signature cysteines residues.) Phylogenetic analysis and genome organization of the chemokine genes showed that successive tandem duplication events generated the CX genes from the CC subfamily. Recombinant CXL-chr24a, one of the CX subfamily members on chromosome 24, showed marked chemotactic activity for carp leukocytes. The mRNA was expressed mainly during a certain period of the embryogenesis, suggesting its role in the zebrafish development.

**Conclusion:**

The phylogenic and genomic organization analyses suggest that a substantial number of chemokine genes in zebrafish were generated by zebrafish-specific tandem duplication events. During such duplications, a novel chemokine subfamily termed CX was generated in zebrafish. Only two human chemokines CXCL12 and CXCL14 have the orthologous chemokines in zebrafish. The diversification observed in the numbers and sequences of chemokines in the fish may reflect the adaptation of the individual species to their respective biological environment.

## Background

Chemokines are a family of chemotactic cytokines that regulate migration and tissue compartmentalization of various kinds of cells in the body [1-4]. Chemokine genes have been found only in vertebrates including jawless fish lamprey, the most primitive vertebrate [5]. Based on the arrangement of the four conserved cysteine residues, chemokines are now classified into four subfamilies, CXC, CC, XC and CX3C. One or three amino acid residues separate the first and second cysteine residues in the CXC and CX3C chemokines, respectively. In the CC subfamily, the two N-terminus cysteines are juxtaposed. The XC subfamily (often called as C subfamily) lacks the first and third conserved cysteine residues. There are at least 44 chemokine genes (24 CC, 17 CXC, one CX3C, and two XC genes) in humans. In addition, there are four CC chemokine genes (CCL3L1, CCL3L3, CCL4L1, and CCL4L2), which are highly similar to CCL3 or CCL4 genes but their gene copy numbers are variable between individuals. In mice, there are 38 genes (21 CC, 15 CXCL, one CX3C, and one XC genes) in addition to several CC genes similar to Ccl19, Ccl21, and Ccl27 with variable copy numbers between strains. Besides gene number differences, there exist lineage-specific chemokine genes as well as genes ambiguous in the orthologous relationship even between human and mouse. On the other hand, only 18 functionally signaling chemokine receptors are identified in both human and mouse. Hence, multiple chemokines can bind to the same receptor.

Chemokine genes can be divided into two groups based on the genomic organization [3]. The genes of one group are located in large clusters at particular chromosome locations (the major-cluster chemokines). In humans and mice, there are two major gene clusters consisting of CC genes and CXC genes, respectively. Another group of chemokines are located separately in unique chromosomal locations, singly or in mini-clusters. The major-cluster chemokines usually bind to multiple receptors and often do not correspond well between species. They were probably generated by multiple gene duplication events occurred relatively recently in the evolutionary terms. In contrast, mini-cluster or non-cluster chemokine genes are well conserved between the species and tend to act on a single receptor.

In addition to the protein or genome structure-based classifications, the chemokines can also be functionally classified into two major groups based on their mode of expression and function, the inflammatory and homeostatic chemokines [6]. Inflammatory chemokines are expressed by leukocytes or related cells only upon activation and mediate emigration of leukocytes, while homeostatic chemokines are constitutively expressed in tissues such as lymphoid tissues and are involved in relocation of lymphocytes or other types of cells. Some chemokines are known to have both properties and are called dual-function chemokines. Notably, most homeostatic and dual-function chemokines belong to the mini- or non-cluster chemokines, while most inflammatory chemokines to the major cluster chemokines.

Teleost fishes constitute an evolutionarily diverse group and form half of the vertebrate species. The availability of genomic resources in the form of genome sequences and ESTs (Expressed Sequence Tags)/cDNAs for fishes that belong to different orders now allows an evolutionary analysis of the chemokine gene family in the fish. The draft genome sequences are available for several teleosts, and we chose zebrafish (*Danio rerio*) and two pufferfish species, *Fugu rubripes *(or *Takifugu rubripes*) [7] and *Tetraodon nigroviridis *[8], for this study. The genome size of zebrafish is 1,700 Mb, about half of the size of human genome, and those of the two pufferfish species are only 390 Mb. The divergence between the lineages leading to zebrafish and pufferfish was estimated to be about 150 million years (Myr) ago from the fossil data [9,10] or about 324 Myr ago from the molecular data [11]. The two pufferfish species belong to the order Tetraodontiformes and diverged from a common ancestor approximately 32 Myr ago from the fossil data [10] or 85 Myr ago from the molecular data [11]. The genome sequences of these fishes are nearly complete, and 85% (zebrafish) and 90% (the two pufferfish species) of the genomes are represented in the draft sequences.

Zebrafish is a useful experimental model to study various biological processes *in vivo*, particularly the developmental processes [12-14]. Previously, by using zebrafish, Doitsidou et al. [15] and Knaut et al. [16] have demonstrated that the CXC chemokine cxcl12a (sdf-1a) and its receptor cxcr4 are critical for proper migration of the primordial germ cells, the progenitors of the gametes. Furthermore, also by using zebrafish, the cxcl12-cxcr4 signaling has also been shown to be important for the migration of sensory cells known as the migrating primordium of the posterior lateral line as well as the guidance of retinal ganglion cell axons [17,18]. These finding have clearly demonstrated the usefulness of this model organism for studying the chemokine functions in the embryogenesis.

Recently, Peatman and Liu [19] reported identification of 46 CC chemokine genes in the zebrafish genome. They compared them with the 26 catfish CC chemokines that they previously identified, and showed evidence of extensive, species-specific intrachromosomal duplications. They also showed such gene duplications are widespread among the teleost fishes by analyzing CC chemokine genes of various species [19]. DeVries et al. [20] also identified 127 vertebrate chemokine genes including 63 zebrafish genes and showed that the tandem and en bloc duplications have been used as means for selective expansion of the chemokine superfamily members. However, the zebrafish genome project is still ongoing, and the genome sequence that these two groups have used was the previous version. In the present study, we systematically searched for the chemokine genes in the latest zebrafish genome and EST databases. We have identified more than 100 chemokine genes in the zebrafish genome including a novel chemokine subfamily termed CX. Phylogenic and syntenic analyses with other fish and mammal chemokines have demonstrated that extensive and highly species-specific duplication events were involved in the evolution of the zebrafish chemokine system. We have further demonstrated that several chemokines including one of the CX subfamily show developmentally regulated expression patterns, suggesting their potential roles in the embryonic development of zebrafish.

## Results

### Chemokine gene prediction and isolation of chemokine cDNAs

Using various chemokine sequences as queries, we searched for chemokine genes in the zebrafish draft genome and EST databases and identified a total of 101 possible chemokine gene sequences (Table 1) in addition to the four chemokine genes reported previously by other groups, ccl1 (GenBank accession number NM_131062), cxcl12a and cxcl12b [15], and scyba [21]. To determine whether the genes predicted from the genome sequence are expressed and also to confirm the exon-intron junctions, a mixture of cDNAs prepared from various developmental stages of zebrafish was used as a template for RT-PCR using primers located outside the coding sequences (Table 2). A total of 34 cDNAs were isolated. The 5' sequence of one clone (CCL-chrUi) was obtained by RACE reaction. Six of them were isolated incidentally due to their close similarity to the original cDNA sequences. Therefore, a total of 107 chemokine genes were newly identified, of which 71 genes have corresponding cDNAs or ESTs (Table 1). Considering the zebrafish draft sequence covers approximately 85% of the genome, these results suggest that there would possibly exist more than the currently identified 111 (4+107) chemokine genes in the zebrafish genome, far exceeding the number of the mammalian chemokine genes. Indeed, there are a number of chemokine-like exons located near or within the intact chemokine genes (not shown). Although most of them may be pseudogenes, the others might turn out to be intact genes if the gaps in the draft sequence are filled by the ongoing sequencing project.

**Table 1:** Chemokine genes in zebrafish^*a*^

**Chemokin**	**Chr**	**Ori**	**Location**	**Exons**^*b*^	**GenBank**	**EST**^*c*^	**aa**^*d*^	**O-Glyco**^*e*^
CCL-chr25a	25	+	19027505–19029332	3	AB331747	EH278089	85	0 (0)
CCL-chr25b	25	+	19035165–19036703	3			110	0 (0)
CCL-chr25c	25	+	19075615–19076539	3			99	0 (0)
CCL-chr25d	25	+	19078908–19089162	3		Dr.112407	96	0 (0)
CCL-chr25e	25	+	19090993–19094737	3			95	0 (0)
CCL-chr25f	25	+	19100444–19101671	3		Dr.89844	102	1 (2)
CCL-chr25g	25	+	19268675–19270505	3		Dr.111511	85	0 (0)
CCL-chr25h	25	+	19276303–19277760	3			109	0 (0)
CCL-chr25i	25	-	19352626–19353853	3		Dr.89844	102	1 (2)
CCL-chr25j	25	-	19359559–19363303	3			95	0 (0)
CCL-chr25k	25	-	19365134–19374852	3			96	0 (0)
CCL-chr25l	25	-	19376636–19377560	3			99	0 (0)
CCL-chr25m	25	-	19421398–19422248	3			201	60 (60)
CCL-chr25n	25	-	19435712–19436261	3			93	0 (0)
CCL-chr25o	25	-	19437496–19438019	3	AB331748	Dr.112677	94	0 (0)
CCL-chr25p	25	-	19457474–19458202	3			157	37 (37)
CCL-chr25q	25	-	19462780–19463308	3			94	1 (1)
CCL-chr25r	25	-	19466008–19467922	3		Dr.125717	87	0 (2)
CCL-chr25s	25	+	19503739–19505640	3	AB331749	Dr.125717	84	0 (1)
CCL-chr25t	25	+	19508322–19508850	3	AB331750		94	1 (1)
CCL-chr25u	25	+	19513230–19513960	3			158	37 (37)
CCL-chr25v	25	+	19536723–19537246	3	AB331751		94	0 (0)
CCL-chr25w	25	+	19538474–19539024	3			93	0 (0)
CCL-chr25x	25	+	19552761–19553847	3			277	104 (104)
CCL-chr25y	25	-	19582718–19584968	3	AB331752	Dr.93495	98	0 (0)
CCL-chr25z	25	-	20857424–20858639	3		Dr.131114	163	36 (36)
CCL-chr25aa	25	-	21213925–21215140	3		Dr.131114	163	36 (36)
CCL-chr25ab	25	-	21241306–21243245	3	AB331753	Dr.93495	98	1 (1)
CCL-chr25ac	25	-	21252391–21254330	3	AB331754	Dr.93495	98	1 (1)
CCL-chr24a	24	+	30502892–30505104	4	AB331755	DV590257	102	3 (3)
CXCL-chr24a	24	-	31852479–31853361	3			187	7 (7)
CXCL-chr24b	24	-	>31853500–31855624	3			153	9 (9)
CXCL-chr24c	24	-	31862084–31862883	3		Dr.92223	159	18 (18)
CXL-chr24a	24	+	32266050–32284596	5	AB331756	Dr.18825	115	0 (1)
CXL-chr24b	24	+	32290478–32291634	5			115	0 (0)
CCL-chr24b	24	+	32293590–32295005	5	AB331757	CO359348	116	0 (1)
CCL-chr24c	24	+	32302418–32304645	5	AB331758	Dr.130538	118	0 (0)
CCL-chr24d	24	+	32306481–32308480	5		CT653601	127	0 (0)
CCL-chr24e	24	+	32322636–32323565	5			118	0 (0)
CCL-chr24f	24	+	32329863–32331934	5			117	0 (0)
CXCL-chr24d	24	+	32345052–32346003	3			210	6 (6)
CXCL-chr24e	24	-	32352898–32353697	3	AB331759	Dr.92223	159	18 (18)
CCL-chr24g	24	-	32414552–32415181	3		Dr.111235	115	0 (0)
CCL-chr24h	24	-	32433297–32434055	4			120	2 (2)
CCL-chr24i	24	-	32440032–32440892	4		Dr.125570	135	17 (18)
CCL-chr24j	24	+	32451758–32453957	4	AB331760	Dr.897	98	0 (0)
CCL-chr24k	24	+	32461305–32462101	4			108	5 (5)
CCL-chr24l	24	+	32463704–32464522	4			106	7 (12)
CCL-chr24m	24	+	32470848–32473296	4	AB331761	Dr.29197	97	0 (2)
CCL-chr24n	24	+	33094971–33096928	5			117	0 (0)
CCL-chr23a	23	+	16185601–16187309	3			245	29 (29)
CXCL12bL	22	-	31836220–31849609	4			97	0 (0)
**cxcl12b**^*f*^	22	-	33091953–33105285	4	NM_198068	Dr.27045	97	0 (0)
CCL-chr20a	20	-	45279550–45280130	3		Dr.22573	103	0 (0)
CCL-chr20b	20	+	455913434–5591923	3		Dr.22573	103	0 (0)
CCL-chr20c	20	+	45594097-45594746	3		Dr.84529	103	0 (0)
CCL-chr20d	20	+	45600362–45600907	3	AB331762	EH279763	101	1 (1)
CCL-chr20e	20	+	45604369–45606327	5		EH547529	174	1 (1)
CCL-chr20f	20	+	45615627–45618919	5			163	3 (3)
CCL-chr20g	20	-	46647358–46648220	4	AB331763		106	0 (0)
CXL-chr19a	19	+	23008696–23011009	5			115	0 (1)
CCL-chr17a	17	-	43053286–43056335	4	AB331764	Dr.110765	111	7 (7)
CCL-chr17b	17	-	43061247–43065632	5		CT653600	127	0 (0)
CCL-chr17c	17	-	>43069244–43070260	(5)^*g*^			108	0 (1)
**scyba**^*f*^	14	-	27560158–27568603	4	NM_131627	Dr.117782	100	0 (0)
CXCL-chr13a	13	+	36660529–36661730	3		EH438452	90	0 (2)
CXCL-chr13b	13	+	36664145–36666791	4		Dr.88	120	0 (0)
**cxcl12a**^*f*^	13	-	36845066–36857673	4	NM_178307	Dr.99352	99	0 (0)
CXCL-chr13c	13	+	51125309–51128037	3	AB331765	Dr.82552	117	7 (8)
CXCL-chr13d	13	+	51138026–51141305	3	AB331766	Dr.92011	101	0 (0)
CXL-chr12a	12	+	23085158–23087450	5		Dr.32443	123	0 (2)
CCL-chr11a	11	+	5493062–5496539	3	AB331767	Dr.81010	106	0 (0)
CCL-chr11b	11	+	5570254–5575583	5	AB331768	Dr.85019	126	1 (2)
CCL-chr10a	10	-	13736505–13739228	4		Dr.92663	114	0 (1)
CCL-chr10b	10	+	(3289242)-48827709	3	AB331769	EE205697	91	0 (0)
**ccl1**^*f*^	8	-	10307292–10323292	3	NM_131062	Dr.76247	93	0 (0)
CCL-chr7a	7	+	51890176–51890837	3		Dr.111629	93	0 (0)
CCL-chr5a	5	-	8165983–8172469	4	AB331770	Dr.86771	119	0 (0)
CCL-chr5b	5	-	8182370–8184709	3		EH281478	107	0 (0)
CXCL-chr5a	5	-	33964121–33965393	4		Dr.84656	115	2 (2)
CXCL-chr5b	5	-	33996816–33998000	4		Dr.111133	102	0 (0)
CXCL-chr5c	5	+	50933627–50940725	4	AB331771	CN175165	147	0 (0)
CXCL-chr5d	5	+	50944403–50945002	4		Dr.115041	94	0 (1)
CXCL-chr5e	5	+	50954047–50954534	4		Dr.89665	121	13 (13)
CXCL-chr5f	5	+	50959591–50960454	4			96	0 (2)
CXCL-chr5g	5	+	51761233–51761767	3			106	2 (2)
CXCL-chr5h	5	+	51766388–51766850	3			92	0 (0)
CXCL-chr5i	5	+	51772479–51772941	3			92	0 (0)
CCL-chr2a	2	-	38292712–38293705	3			247	47 (47)
CCL-chr2b	2	-	38310649–38315607	3			195	16 (16)
CCL-chr2c	2	-	38329197–38329990	3		Dr.113953	150	0 (0)
XCL-chr2a	2	-	38339304–38339976	3		EH453597	126	1 (3)
CCL-chr2d	2	+	38353445–38354111	4		Dr.86775	138	8 (8)
CCL-chr2e	2	+	38356664–38358727	4	AB331772	Dr.113952	111	4 (4)
CCL-chr2f	2	-	38369684–38371438	3		Dr.92436	89	0 (0)
CXCL-chr1a	1	+	14796681–14797310	4			98	0 (0)
CXCL-chr1b	1	+	15001965–15002594	4			98	0 (0)
CXCL-chr1c	1	-	61228824–61231793	4	AB331773	Dr.17289	114	0 (1)
CCL-chr1a	1	-	68219709–68220766	3			254	59 (59)
CCL-chr1b	1	-	68251400–68252095	3			130	0 (0)
CCL-chr1c	1	-	68253292–68254202	3			220	26 (26)
CCL-chrUa	U				AB331774	EH599800	99	0 (0)
CCL-chrUb	U				AB331775		108	0 (0)
CCL-chrUc	U				AB331776		99	0 (0)
CCL-chrUd	U				AB331777		94	1 (1)
CCL-chrUe	U				AB331778		110	0 (0)
CCL-chrUf	U					Dr.83347	144	6 (6)
CCL-chrUg	U					CK237158	131	3 (3)
CCL-chrUh	U					EH479986	95	0 (0)
CCL-chrUi	U				AB331779	BQ615577	93	0 (0)
CCL-chrUj	U				AB331780		86	0 (0)

*a *Zebrafish Zv6 assembly was used for the analyses.

*b *Number of exons containing coding sequences.

*c *Dr numbers are Unigene IDs (Build #101).

*d *Amino acid residues. e Number of O-glycosylation sites in the COOH-terminal region. The number in parentheses shows such number in the whole mature peptide.

*f *Bold letters are approved gene symbols.

*g *The last exon sequence has not yet been determined.

**Table 2:** PCR primers used for isolation of zebrafish chemokine cDNAs

**Chemokine**	**Forward primer**	**Reverse primer**
CCL-chr25a	5'-CATCTGCAAGAGATCACAAATC	5'-GGACAGTTGCATTAAGCAGAAA
CCL-chr25o	5'-CATCTCCTGATTCCTTCTGTGA	5'-GGTCAAACATATGATGAAGCACA
CCL-chr25s	5'-CAGCAGAGGAGCTGTGAATG	5'-AGAATACAAAATTGCCTTTTCCT
CCL-chr25t	5'-TCCTGATTCCTTCTGTGATTGA	5'-CACATCAGAAATAGTACAAAAGGTCA
CCL-chr25v	5'-CATCTCCTGATTCCTTCTGTGA	5'-GGTCAAACATATGATGAAGCACA
CCL-chr25y	5'-CACACATTAGATCCAGCTTGAGA	5'-GGTTCAGAAGTTAGAGTACGCAGA
CCL-chr25ab	5'-CACACATTAGATCCAGCTTGAGA	5'-GGTTCAGAAGTTAGAGTACGCAGA
CCL-chr25ac	5'-CACACATTAGATCCAGCTTGAGA	5'-GGTTCAGAAGTTAGAGTACGCAGA
CCL-chr24a	5'-CACTTTCAAGATGGGCAACA	5'-GAGTTTTAGCCTTAAGATAAAGTGGTG
CXL-chr24a	5'-TGTGAAATCCTTCAGCAAACA	5'-TTCACAGATGTCCAGTTTCCA
CCL-chr24b	5'-TCATTCAAGAAATCATTCAGCAA	5'-TGAAAATTTCTTTCCGGTGTG
CCL-chr24c	5'-TCTGTGAAATCATTCAGCAATC	5'-GAAGTTCTCCGATGTCCAGTC
CXCL-chr24e	5'-TGGACGACAAACAGACTCACA	5'-CAGCATATTTTATAGCATAATGTTCAA
CCL-chr24j	5'-CAGCTGACCATCACCAAACA	5'-AGTTTTGCAGCATCTTCAAGG
CCL-chr24m	5'-TCAGATACCAGAAGTGAAACTGTGA	5'-TGGTGGTATCGTGGAAGTCA
CCL-chr20d	5'-GATCAGCCACCGATAAACCT	5'-ACCAGCAGAGCGTTCTCATC
CCL-chr20g	5'-GCATCAAGATCTGCTCATCG	5'-TGAGAAACGATTCAGTGCAA
CCL-chr17a	5'-CAAAACATGATGAGTATTTGTTGGA	5'-GGAATCATCACTGTCAACTTGC
CXCL-chr13c	5'-AGCATCTTCACCTCTTGTATGC	5'-TTTTGTTGTGCCGAATCTCA
CXCL-chr13d	5'-TCTTCAGCCATCAAGAACGTC	5'-TCTCCTGCAGTGAAACGATG
CCL-chr11a	5'-TCTTCCCAGAACCTCTGAGC	5'-GTTTGTGCATTAAATCAAACGA
CCL-chr11b	5'-GACAGCACGGAGGAAAAATC	5'-TGACACACAAGAAGGCTGGA
CCL-chr10b	5'-GAAATCACCCCGATCTGCT	5'-AGGATCAACACGAGCAAACA
CCL-chr5a	5'-CGATAGAGCGGTGAGAGGAG	5'-TTAGCATAAACAGCTGCCATT
CXCL-chr5c	5'-GTTTCATGCAGGGATTTTCC	5'-TTGTGACATCACGCGGAGGA
CCL-chr2e	5'-TGAGCATCTCTACATCTTTGCAT	5'-AGAGGTATGGAGTCAGTGCTGT
CXCL-chr1c	5'-TCAGCAGCACTTCATTCAC	5'-CAGTGTACAGCGAGCGAATC
CCL-chrUa	5'-CAAAACATGATGAGTATTTGTTGGA	5'-GGAATCATCACTGTCAACTTGC
CCL-chrUb	5'-CAAAACATGATGAGTATTTGTTGGA	5'-GGAATCATCACTGTCAACTTGC
CCL-chrUc	5'-CAACTGGTTCATCAAGCATACC	5'-GGGGTATGTATCCAAGTAAGTGC
CCL-chrUd	5'-CATCTCCTGATTCCTTCTGTGA	5'-GGTCAAACATATGATGAAGCACA
CCL-chrUe	5'-CATCAACCACACCAGCCTTA	5'-AAAGGACAGTTGCATTAAGCAGT
CCL-chrUi	5'-TCTTGAGAAGCAGAATCAGCAG	5'-CAAAGATCATCACCACATCTGAA
CCL-chrUj	5'-CAGCAGAGGAGCTGTGAATG	5'-AGAATACAAAATTGCCTTTTCCT

The 111 chemokine genes found in zebrafish represents a dramatic increase when compared to the total number of chemokine genes in human or mouse [3]. To determine whether such an expansion of the chemokine genes occurred in other teleosts, we also searched chemokine genes in the genomes of two other teleost fishes, fugu (*Fugu rubripes*) and its close relative *Tetraodon *(*Tetraodon nigroviridis*). Both fugu and *Tetraodon *have a compact genome of 390- and 350-Mb, respectively, and 90% of each genome is covered by assembled sequences. Contrary to zebrafish, each pufferfish contains only 18 chemokine genes (see Additional file 1). Of them, 15 genes are apparently the orthologous genes between the two pufferfishes.

The zebrafish chemokine genes identified in this study were compared with those recently identified by two other groups, DeVries et al. [20] and Peatman and Liu [19] (Additional file 2). Fugu chemokines reported by DeVries et al. [20] were similarly compared (Additional file 2). The lists of the zebrafish chemokine genes made by these two groups also included partial genes. We felt it necessary to exclude most of the partial chemokine genes for the following analyses to avoid pseudogenes except when the missing first or last exons of the partial genes are apparently present in the unsequenced gapped regions. Therefore, some chemokine genes reported by the two groups are not listed in the additional file. Furthermore, genes with different exonintron splicing junctions or genes that are similar but not identical to the ones identified in the present study are shown in parentheses in the file.

### Nomenclature

Since no clear orthology to the mammalian chemokines could be established for most of the fish chemokines (see below), genes identified in this study were named as follows. Zebrafish chemokine genes were designated according to their subfamily + L standing for ligand (CCL, CXCL, XCL, or CXL), followed by the chromosome number prefixed with chr and by alphabets to distinguish individual genes on a given chromosome. For example, CCL-chr25a stands for one of the CC chemokine ligand genes found on chromosome 25. When the chemokine genomic locus of an EST or cDNA-derived chemokine gene was unknown, 'U' was used instead of the chromosome number. Pufferfish chemokine genes were similarly termed. Since the genome of fugu is organized into scaffolds that are not yet organized into chromosomes, *Tetraodon *chromosome numbers were used for fugu nomenclature. For the fish genes that have already approved gene symbols or names termed by other researchers, they are called by their symbols or given names.

### Protein structural characteristics of fish chemokines

There are 81 CC, 25 CXC, and one XC subfamily member genes in the zebrafish genome, but no CX3C subfamily member has been identified. Mammalian CXC chemokines with neutrophil chemotactic activity often contain the ELR motif (a conserved Glu-Leu-Arg sequence preceding the first cysteine) [22], but no zebrafish CXC chemokines are found to have this motif. Although zebrafish possesses one XC subfamily member, XCL-chr2a, it shows little homology to the mammalian XC chemokines.

Two membrane-bound chemkines, CX3CL1 and CXCL16, are known in the mammals [3]. To explore fish chemokines with potential transmembrane domains, the chemokine sequences were analyzed with SOSUI. Three zebrafish chemokines, CXCL-chr24a, CXCL-chr24b, and CXCL-chr24d, were revealed to have one potential transmembrane segment in the COOH-terminal region (see Additional file 3). Compared to the other chemokines that are approximately 100 amino acids (a.a.) long, these three chemokines are much longer (Table 1), and the transmembrane segments are located at positions 133–155 of CXCL-chr24a (187 a.a. long), 132–153 of CXCLchr24b (153 a.a. long), and 133-155 of CXCL-chr24d (210 a.a. long).

Just like the mammalian transmembrane chemokines, CX3CL1 [23] and CXCL16 [24], the three zebrafish chemokines with a potential transmembrane region also have Ser/Thr-rich mucin-like domains between the chemokine domain and the putative transmembrane region (Table 1). Their mucin domains tether and extend the chemokine domains away from the cell surface, allowing their efficient interaction their receptors expressed by target cells [25,26]. Of note, more than one third of the zebrafish chemokines with no apparent transmembrane region are also found to have potential mucin-type O-glycosylation sites in the COOH-terminal region (Table 1). Especially, CCL-chr25x has more than 100 possible O-glycosylation sites in the Cterminal region downstream from the fourth conserved cysteine residue. On the other hand, the pufferfishes have few such chemokines like mammals (see Additional file 1).

### Zebrafish has a novel chemokine gene subfamily CX

**Figure 1:**
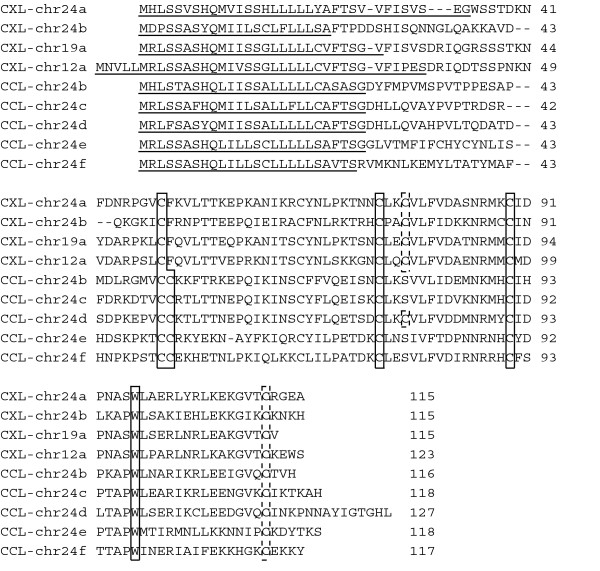
**Amino acid sequences of zebrafish CX chemokines**. Sequences were aligned with CLUSTAL W. The five CC chemokines closely related to the CX chemokines are also shown. Solid boxes indicate the conserved four cysteine residues and the tryptophan residues often found seven a.a. downstream from the fourth cysteine residue. Dotted boxes denote the additional cysteine residues conserved among CX chemokines. Predicted signal sequences are underlined.

We found four possible zebrafish chemokines, CXL-chr12a, CXL-chr19a, CXLchr24a, and CXL-chr24b, that miss one of the two N-terminus conserved cysteine residues and thus retain only three signature cysteine residues (Figure 1). (Note that the XC subfamily lacks the first and third conserved cysteine resides.) We designated the group of the genes as a novel CX chemokine subfamily because two of the member genes (CXL-chr24a and CXL-chr24b) are clustered with other CC chemokine genes on chromosome 24, and one of them (CXL-chr24a) shows a chemotactic activity (see below). Our database search showed that this novel class chemokines does not exist in other species. Although Gilligan et al. [27] reported identification of a similar CX chemokine fCL1 in the fugu genome, our analysis of the recent fugu draft genome revealed that it is actually a CC chemokine (see Additional file 1).

These novel CX subfamily genes show low similarities to other chemokines, but contain a tryptophan residue at the seventh position downstream from the fourth conserved cysteine residue often found in other chemokines (Figure 1). Furthermore, there are three additional cysteine residues that are specifically conserved among the four CX members (Figure 1). Although we isolated one cDNA clone for CXL-chr24a, there are two EST records for CXL-chr12a. There are, however, no expression data available for the other two CX members.

### Phylogenetic analysis of the chemokine genes

**Figure 2:**
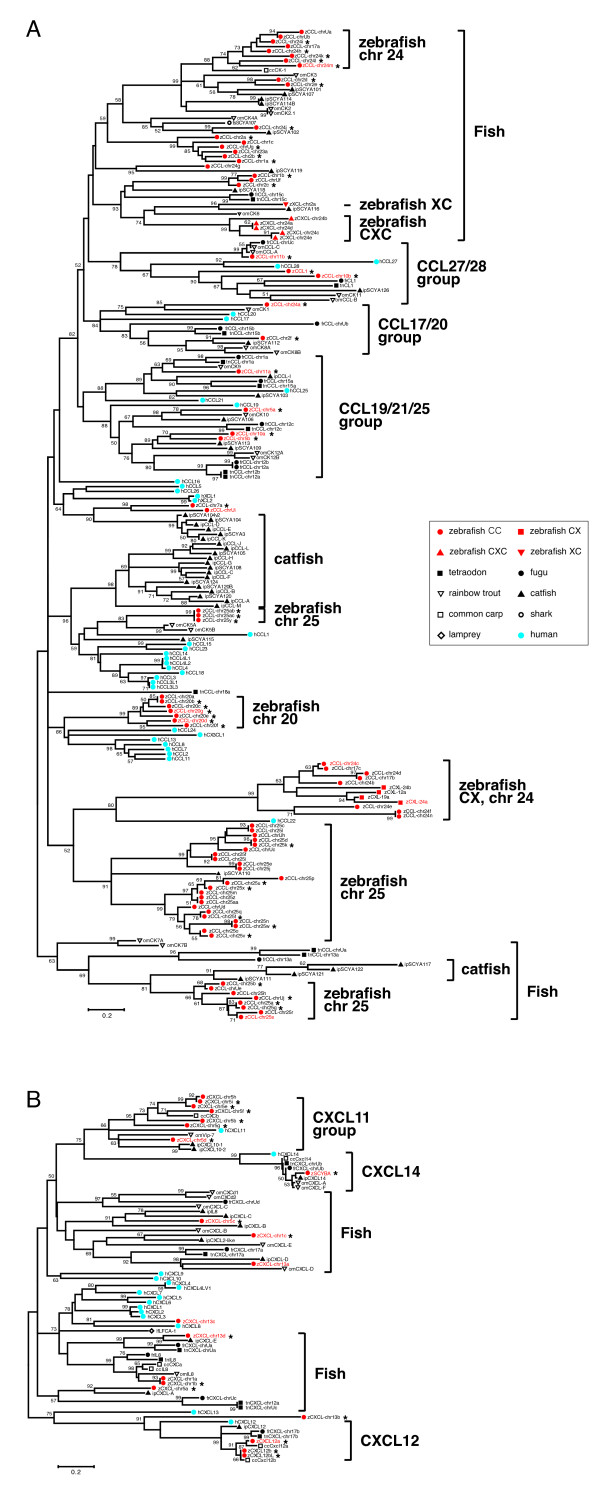
**Phylogenetic analyses of fish and human chemokines**. Since a large number of chemokines were analyzed, trees were separately constructed with two groups of chemokines (**A **and **B**) using the Dayhoff matrix and the neighborjoining method. Four subfamilies of zebrafish chemokines and other species chemokines are indicated by colors and symbols. The zebrafish chemokine genes used in the RT-PCR analyses (Figure 5) are shown in red letters, and the genes identical or similar to those identified by DeVries et al. [20] and Peatman and Liu [19] are shown by asterisks (see also Additional file 2). European river lamprey (*Lampetra fluviatilis*, jawless fish) CXC chemokine LFCA-1 and shark (*Triakis scyllium*, elasmobranch fish) CC chemokine SCYA107 were also included. Numbers at branch nodes represent the confidence of interior-branch test with 1000 iterations. Only confidence values of ≥50% are shown. The nodes of large branches are unreliable in most cases due to the low values. Amino acid sequences of zebrafish and pufferfish chemokines are shown in Additional file 3. Abbreviations: z, zebrafish; fr, fugu; tn, *Tetraodon*; h, human; ip, channel catfish; om, rainbow trout; lf, European river lamprey; ts, shark.

To analyze the evolutionary relationships between the fish and human chemokines, a phylogenetic tree was constructed. For this analysis, the chemokine sequences from three more fish species, channel catfish (*Ictalurus punctatus*), rainbow trout (*Oncorhynchus mykiss*) and common carp (*Cyprinus carpio*), were also included. Their sequences were retrieved from the literatures [28-35] and from the EST databases. Preliminary construction of a phlylogenetic tree using all the sequences showed two large clusters (see Additional file 4). One cluster was mainly comprised of CC, XC, CX, and CX3C subfamily members of all species. Interestingly, five zebrafish CXC chemokine genes on chromosome 24 were also included in this group. The other cluster contained only CXC chemokines from various species. Each cluster in the tree forms a monophyletic clade supported with a high confidence value. However, in order to obtain a more reliable tree, a phylogenetic tree for each cluster was constructed separately since a large number of structurally divergent sequences were used. Figure 2 shows the trees of the two clusters using the JTT matrix for the amino acid alignment. Dayhoff's (PAM) matrix was also tested for each alignment, but the results were essentially the same (not shown).

To root the phylogenetic trees, CXC chemokine LFCA-1 of jawless fish river lamprey or CC chemokine SCYA107 of elasmobranch fish shark was added in the tree construction as an outgroup. Unexpectedly, these two chemokines are very closely related to teleost fish chemokines (Figure 2), suggesting that the chemokine family have already existed prior to the divergence of lamprey and other organisms. Therefore, those chemokines were not used as an outgroup.

**Figure 3:**
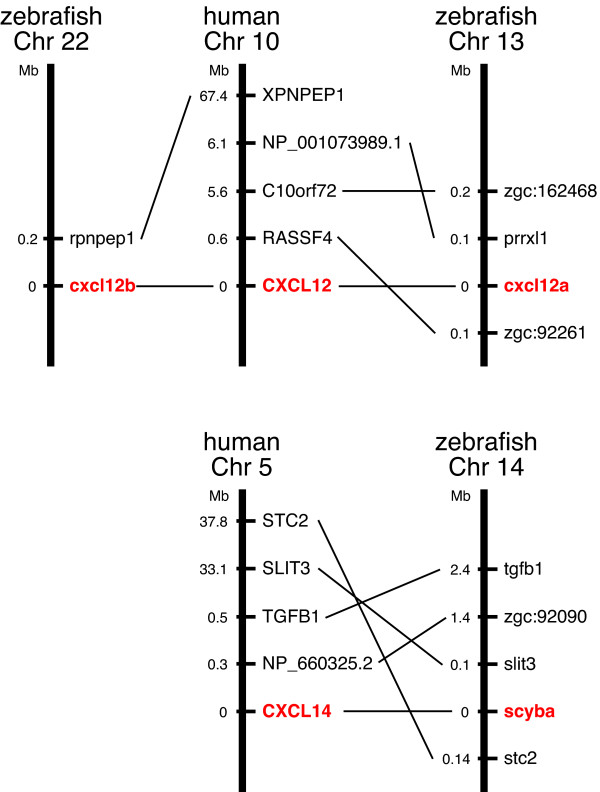
**Diagram of synteny of genes between human and zebrafish CXCL12 and CXCL14 loci**. Human CXCL12/zebrafish cxcl12a and cxcl12b genes and human CXCL14/zebrafish scyba genes are shown in red letters.

The majority of the fish chemokines form their own branches that do not include human chemokines, suggesting that most fish chemokines have been generated after the divergence of fish and mammals. A few orthologous relationships between fish and human chemokines are inferred based on the phylogenetic trees generated by using two matrices (Figure 2). However, based on the synteny analyses, we have identified only two definitive orthologous relationships. Zebrafish cxcl12a and cxcl12b are found to be orthologous to human CXCL12, and unambiguous 1:1 orthology is observed between zebrafish scyba and human CXCL14 (Figure 3). Human CXCL12 and *Tetraodon *CXCL-chr17b are also identified as the orthologous genes based on the phylogenetic (Figure 2B) and synteny analyses (not shown). Huising et al. (2003) [34] and [20] also found that only CXCL12 and CXCL14 have unambiguous orthologues in fish.

Previously, by constructing a phylogenetic tree using a collectioin of CC chemokines from four teleost species including zebrafish, Peatman and Liu have observed four highly related chemokine groups, CCL19/21/25, CCL20, CCL27/28 and fish-specific groups, in the tree [36]. However, the bootstrapping support of these groups is low, and some groups are quite large and consisting of several branches. Our phylogenic trees also demonstrate groups such as CCL17/20, CCL19/21/25, CCL27/28 and fishspecific groups (Figure 2A). In the fish-specific groups of our tree, there are several subgroups containing only zebrafish or catfish chemokines. Notably, such zebrafish chemokines are located on the same chromosomes, suggesting their species-specific amplification by tandem duplication (see below). Although Peatman and Liu also described the presence of large MIP and MCP CC chemokine groups in their tree [36], such groups are not found in our tree most probably due to the usage of different alignment and tree construction methods. In the CXC chemokines, one CXCL11 group and two fish-specific groups are evident in addition to the orthologous groups of CXCL12 and CXCL14 (Figure 2B). Furthermore, the chemokine genes reported by DeVries et al. [20] and Peatman and Liu [19] are clearly found within each group of chemokines. Thus, the CX group and neighboring CC genes on chromosome 24 only identified in the present study constitutes truly a novel gene cluster in the zebrafish genome.

### Genome organization of the zebrafish chemokine genes

**Figure 4:**
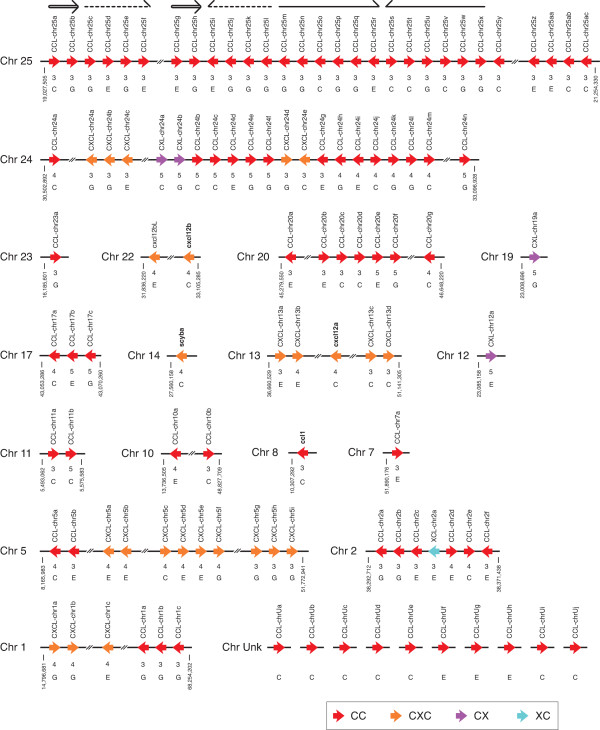
**Schematic genomic organization of the zebrafish chemokine gene family**. Arrowheads indicate chemokine genes and their transcriptional orientation, and four subfamilies of zebrafish chemokines and other species chemokines are colored as in Figure 2. The numbers under the chemokine genes indicate the numbers of coding exons. The alphabetical letters under the exon numbers show: G, genes predicted only from genomic data; C and E, cDNA clones or EST data available for the genes. Arrows above chromosome 25 show direct or inverted repeats. Dashed lines denote spaces of more than 100 kb.

The zebrafish chemokine genes are distributed on 17 different chromosomes, but 10 genes have not yet been localized on any chromosomes. There are two large chemokine gene clusters in the zebrafish genome, located on chromosomes 24 and 25 containing 29 and 21 genes, respectively (Figure 4). Other genes are present in miniclusters or as single genes on 15 different chromosomes. In contrast to the mammalian chemokine gene organization, genes of different subfamilies, CC, CXC, XC, and CX, are co-localized in a large cluster on zebrafish chromosome 24, even though the largest cluster on zebrafish chromosome 25 consists of only CC subfamily genes. The single zebrafish XC chemokine gene, XCL-chr2a, is located within a mini-cluster of CC genes on chromosome 2, while the mammalian XC chemokine genes are located separate from other chemokine genes. When the genome organization is compared with the phylogenetic tree (Figure 2), zebrafish genes consisting of the same major or mini-clusters also tend to locate in the same branches of the phylogenetic tree. For example, the majority of the CC chemokine genes from the major clusters on chromosomes 24 and 25 form separate branches consisting of only zebrafish genes in the phylogenetic tree. Similarly, the CXC and CC mini-cluster genes on chromosomes 5 and 20 form separate branches, respectively. These results suggest that the cluster genes arose from extensive tandem duplication events following the divergence of zebrafish and other teleosts. In the catfish genome, tandem duplications might have also occurred on some chromosomes as three branches consisting of only catfish CC chemokines are present in the tree (Figure 2A). In addition, when the major cluster on chromosome 25 is analyzed by dot-plot analyses (not shown), three direct or inverted repeats, each containing several chemokine genes, are found (Figure 4). Most of the genes in one of the repeat units are identical or more than 90% similar to the corresponding genes in other repeat units (Additional file 5). Collectively, these genome structures strongly suggest that relatively recent segmental duplication events were involved in the expansion of the zebrafish chemokine genes.

As mentioned above, the major cluster on zebrafish chromosome 24 contains three different subfamily members. The five CXC genes in the cluster form a separate branch in the phylogenetic tree along with other CC chemokine branches. Similarly, two CX genes in the gene cluster (CXL-chr24a and CXL-chr24b) and two other CX genes on other chromosomes (CXL-chr19a and CXL-chr12a) form a separate branch with some CC genes on chromosome 24 in the tree. These results suggest that these CXC and CX genes were generated from CC genes in zebrafish.

A close inspection of the CX and the neighboring CC genes on chromosome 24 has further supported their derivation from a common ancestor gene. First, although the number of coding exons in the chemokine genes is generally three or four, the coding sequences of the CX and the five neighboring CC genes on chromosome 24 are split into five exons (Figure 4). Second, the neighboring CC chemokines also contain two or three of the three extra cysteine residues specifically conserved among the CX subfamily (Figure 1). These common structural features indicate that the two CX genes on the chromosome 24 were generated in the zebrafish genome by successive tandem duplication events from the neighboring CC chemokines with five exons. Of note, two CC genes on chromosome 17, CCL-chr17b and CCL-chr17c, also contain one or three of the additional cysteine residues (see Additional file 3) and consist of five coding exons (Figure 4). Furthermore, both genes are in the same branch in the phylogenetic tree with the CX genes (Figure 2A). These features suggest that one of the duplicated CX immediate ancestor genes have translocated to another chromosome.

### Developmentally regulated zebrafish chemokine gene expression

Zebrafish embryonic development is rapid [12]. At about two days post fertilization all common vertebrate specific body features can be seen, including a compartmentalized brain, eyes, ears, and all internal organs. The zebrafish larvae are able to swim as early as five days after fertilization and become adults at around three months later. Macrophages have been identified in zebrafish as early as 15 hours post fertilization (hpf) [37], and neutrophilic granulocytes circulate by 48 hpf [38,39].

**Figure 5:**
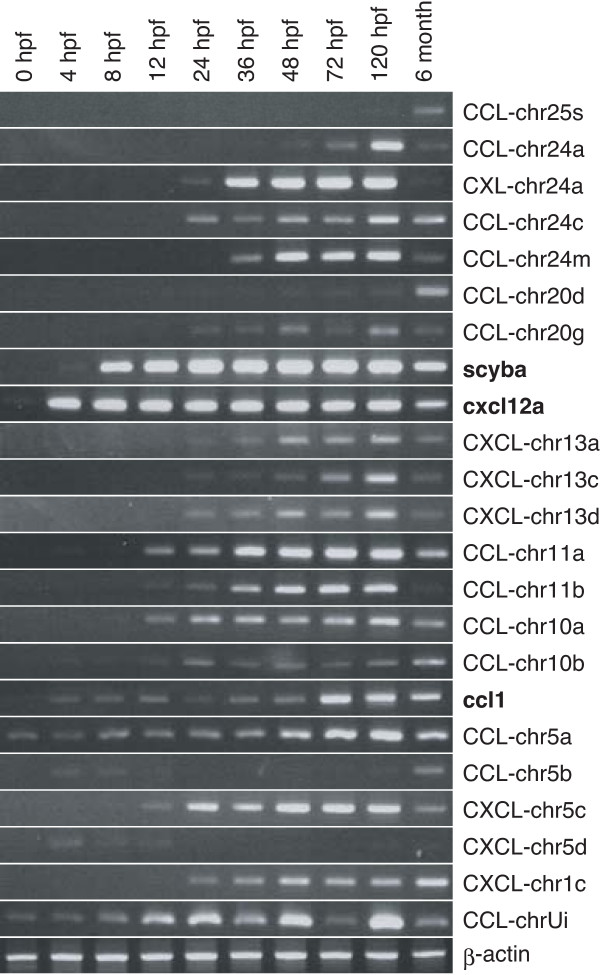
**5 RT-PCR analyses of chemokine expression pattern in zebrafish embryonic developmental stages**. PCR reactions were performed using cDNAs prepared from embryos at various developmental stages. Primers used are listed in Table 3. β-actin was used as an internal control.

In order to investigate the gene expression pattern of zebrafish chemokines in the early embryonic developmental stages, we performed semi-quantitative RT-PCR analysis using primers for 23 representative chemokine genes (Figure 5). These genes were selected based on the sequence similarity to other member genes, and indicated with red letters in the phylogenetic trees (Figure 2). The highly similar genes were excluded from the present analysis, and the primers (Table 3) were carefully designed not to cross-amplify other genes. However, we can not formally exclude the possibility that there exist undiscovered genes that closely resemble to any of the selected genes. Three previously reported chemokine genes, ccl1, cxcl12a, and scyba, were also included.

**Table 3:** Primers used for RT-PCR

**Chemokine**	**Forward primer**	**Reverse primer**	P**roduct size (bp)**
CCL-chr25s	5'-TTCTGCTGTTTTTGGTGTTCA	5'-GCTTTATCCAGTCCTCATCTGG	216
CCL-chr24a	5'-TACTTCTCAGCTTCCTTGTGGAGA	5'-CTCATCGTCTTCGTCTTCATTGTT	250
CXL-chr24a	5'-AGGTTGGAGCAGTACCGACAA	5'-CCTCCCCTCTACACGTTACCC	245
CCL-chr24c	5'-ATGCGTCTGTCCTCAGCTTT	5'-GCTGTCGGATCTATGCAGTG	284
CCL-chr24m	5'-AGGAGTTTGGCTGTTGCACT	5'-CCTCCTCACCCACAGTTGTC	219
CCL-chr20d	5'-CAGACAAGTGCTGCTTTTCG	5'-GAGCTGGCCTCTGTGTCTTT	209
CCL-chr20g	5'-CGCTTTCTGCTCAGTTGTTG	5'-TCAGTCTCCGAACCCATTTC	215
**scyba***a*	5'-TGCAGATGCACAAGAAAAGG	5'-GGCTTCAAACGTCCTGTGTT	225
**cxcl12a***a*	5'-TTCATGCACCGATTTCCAAC	5'-TGTTGATGGCGTTCTTCAGG	222
CXCL-chr13a	5'-CAACATGTCCCAAAGACCTG	5'-TTACCTGTTCCAACATTTCATTAGA	201
CXCL-chr13c	5'-ACTGCCCATCCAGCAGTTAC	5'-TCCTGCCTTCGATGATCTTT	200
CXCL-chr13d	5'-TTGTTTTTGGAGTCAGCATCA	5'-AGTGTGTTCTGGGGTTCAGG	222
CCL-chr11a	5'-CCAGCATCGCACAAGGTTAC	5'-GCCTGGACCCAGTCACTTCT	208
CCL-chr11b	5'-ATGCAGCGAGATGTTCAGTG	5'-GGAATTCACAACAGCGACCT	282
CCL-chr10a	5'-CGATGATGCAGTGGATTGTT	5'-TTTCCTGGGTCGTTTTTGTG	235
CCL-chr10b	5'-TACTGTGCATCGCCTTCAGC	5'-TAGACGAGCCATTCGCTTCA	233
**ccl1***a*	5'-GGCACGAGATGGAGTTCAGA	5'-GCCGTCTGTATCCGTGATTT	329
CCL-chr5a	5'-ACGGTCAGCCCAAGAGTCAT	5'-GAGCATGACAGCGTCTCTCG	229
CCL-chr5b	5-CCATAACTCTCCTGCTCATCG	5'-GCGCAGAGTTTCTTGTCCTT	202
CXCL-chr5c	5'-AAGTGCGTGCCTGATAAACC	5'-CACATCAACTGCCTGAATGG	229
CXCL-chr5d	5'-TGTTACTGCTCTGCTTCTTGTCA	5'-CATCGTTTTCCAGCTCCATT	204
CXCL-chr1c	5'-TACGTCCAGCAGGGAGAAGT	5'-CCCTGCTGTTTTTGTGGATT	218
CCL-chrUi	5'-GACTTCTGGCCGTCACATTC	5'-AGCTGAGGGTCCACACAGAG	202
β-actin	5'-TGTTCGAGACCTTCAACACC	5'-TGAAGGTGGTCTCGTGGATA	471

*a *Bold letters indicate approved gene symbols.

Most of the examined chemokine genes started to be expressed at the specific embryonic stages and their expression continued until six months after fertilization. However, there are some exceptions. The transcripts of CCL-chr25s, CCL-chr20d, and CCL-chr5b were expressed mainly in adults, whereas those of CXL-chr24a and CCL-chr11b were detected only at certain developmental stages, i.e., 36–120 hpf. The expression levels of the most genes examined were gradually increased during the embryonic (4–48 hpf) and early larval (72–120 hpf) stages, especially CCL-chr24a and CXCl-chr13c being almost exclusively expressed at early larval periods. Interestingly, the CCL-chr5b was expressed temporarily between 4 and 12 hpf albeit at low levels, and again in the adult stage at much higher levels. In contrast, CCL-chr5a, CCLchrUi, cxcl12a, and ccl1 were expressed throughout the developmental stages from 0 hpf to adults, although their expression levels in eggs at 0 hpf were quite low compared to those of the other embryonic stages. These results suggest that most of the chemokines tested might have some roles during the zebrafish development.

### Zebrafish chemokine CXL-chr24a has a chemotactic activity

**Figure 6:**
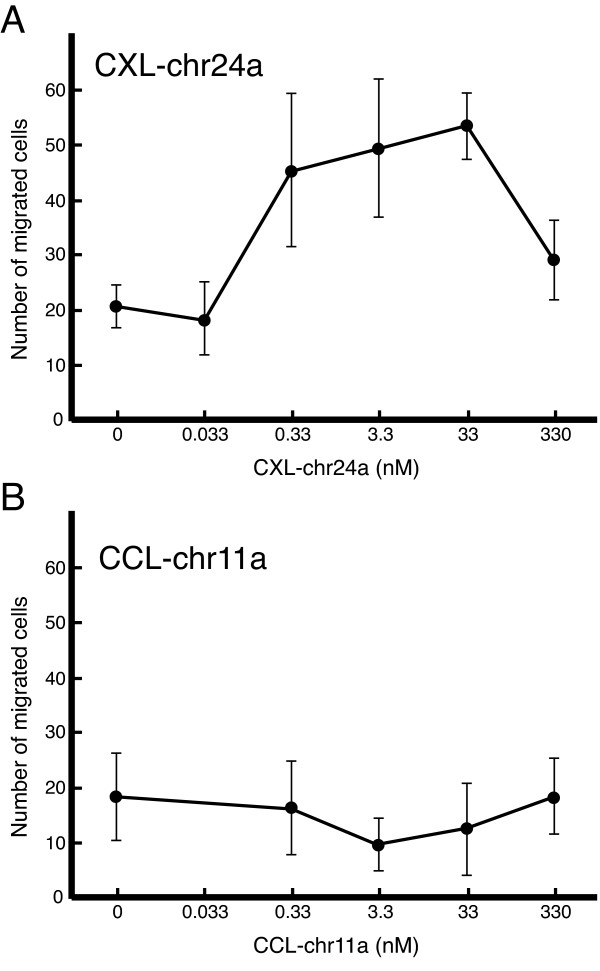
**Chemotactic activity of zebrafish CXL-chr24a and CCL-chr11a for carp leukocytes**. Recombinant CXL-chr24a (A) and CCL-chr11a (B) were tested for dose-dependent chemotactic activity (microchamber assay) on carp leukocytes. Chemotactic responses are expressed as the mean numbers of cells per 0.4 mm^3 ^± SD (n = 3).

CCL-chr11a and CXL-chr24a with a (His)_6 _tag at the C-terminus were expressed in HEK293 cells and purified from the culture supernatants with a His-binding Niaffinity column. To test the chemotactic activity of the recombinant proteins, leukocytes were prepared from the common carp, which belongs to the same family, *Cyprinidae*, as zebrafish. The phylogenetic tree also indicates the close relativeness of both fishes (Figure 2). Recombinant CXL-chr24a showed a potent chemotactic activity for leukocytes from 0.033 to 33 nM (Figure 6A). Like other chemotactic molecules, the activity was decreased at higher ligand concentrations. The attracted cells were mainly neutrophils and lymphocytes (not shown). In contrast, recombinant CCL-chr11a showed little chemotactic activity for carp leukocytes (Figure 6B). This may be due to the lack of receptor expression or weak cross reactivity by carp leukocytes.

## Discussion

Our previous analyses of human and mouse chemokine gene clusters have shown substantial species differences, including species-specific expansions of gene subsets, gene deletions, and generation of a fusion gene [40-43]. Our analysis of old world monkey genome have also revealed that chemokine gene clusters are the subject of frequent birth-and-death events even after the divergence of primates [44]. Recent completion of the chimpanzee genome sequencing revealed that the chemokine family is one of the most rapidly evolving gene clusters [45]. Our present analyses also show that the chemokine family has expanded its member genes in zebrafish due to extensive gene duplication events after the divergence of zebrafish and other teleosts.

In sharp contrast to the large number of genes for the ligands of the chemokine superfamily in zebrafish, our preliminary search has revealed only 26 putative chemokine receptor genes in the zebrafish genome (not shown). DeVries et al. also identified only 24 chemokine receptor genes in zebrafish [20]. This large gene number difference between the ligand and receptor genes might indicate that chemokine genes had duplicated more rapidly than the receptor genes. However, since the chemokine receptor gene family belongs to the large G protein-coupled receptor (GPCR) superfamily, it is difficult to distinguish chemokine receptor genes from other GPCR genes in some cases. Therefore, the receptor gene number in the zebrafish genome might be underestimated, and there is a possibility that the fishspecific chemokines, particularly the CX chemokines, may bind to receptors substantially different from other chemokine receptors and thus may be difficult to identify by the standard database search.

Duplication of genes is believed to be the major force in the evolution of eukaryotic organisms and increases the diversity in expression and function [46]. In zebrafish, extensive duplication events have occurred on some chromosomes and generated a novel subfamily CX and some CXC member genes from the neighboring CC genes. Such duplications increased the number of chemokine genes, particularly inflammatory genes, and might have provided a survival advantage to zebrafish, which is a fresh water fish in temperate regions and possibly confronted with a constant threat of invasion by infectious agents. Another fresh water fish, catfish, might have recently expanded its chemokine gene members by duplication since catfish chemokines tend to form separate groups in the phylogenetic trees (Figure 2). In sharp contrast, pufferfish, a marine or brackish water fish, might have much less chemokine genes in part due to an alternative strategy for their survival and in part due to their dwelling environment. Recently, the draft genome sequence for Medaka (*Oryzias latipes*) has been reported [9]. The genome size of Medaka is about 700 Mb, the size intermediate between those of zebrafish and pufferfish, and Medaka diverged from the pufferfish lineages about 98 Myr ago by the fossile data [10] or 191 Myr ago by the molecular data [11] after the split of zebrafish. It is therefore interesting to see how many chemokine genes the Medaka genome contains and also how well the chemokine genes are shared between Medaka and zebrafish.

Not all chemokine genes have undergone rapid evolution. Some human and fish chemokine genes are orthologous based on the phylogenetic trees and syntenic relationship, indicating that they existed prior to the split of fish and mammals (Figure 2). Their human orthologs, CXCL12, and CXCL14, are known as the homeostatic chemokines that have pivotal roles in the development and the maintenance of the functional integrity of the organism. Since the genes have been conserved throughout the evolution and are thus more close to ancestral genes, they are not located in the major gene clusters and belong to the mini- or non-cluster chemokines in both zebrafish (Figure 4) and mammals [3]. In addition to these orthologous genes, several gene groups that include human genes are observed in the trees (Figure 2). Most human genes in such groups are homeostatic chemokines. However, chemokines in these groups cannot be categorized as orthologous and are not located in the syntenic loci. Therefore, their genes might have been derived from common ancestor genes and are the paralogous genes generated by duplications, but their true orthologous genes might have been drifted in sequence too much or even deleted during evolution. Alternatively, the true orthologous genes or ESTs have not been sequenced yet. It has been shown that a substantial number of chemokine genes in the chicken genome had already existed before the divergence between aves and mammals [47]. Therefore, a considerable number of chemokine genes might have been lost in fish after the divergence of fish and the lineage leading to aves and mammals.

Genome analyses of teleosts including fugu and zebrafish have led to a hypothesis that a whole-genome duplication occurred in the teleost lineage 370 Myr ago during the early evolution of teleosts [8,48,49]. One example of the genes generated by the whole-genome duplication may be cxcl12a gene on chromosome 13 and cxcl12b or cxcl12bL genes on chromosome 22. However, most of the expansion of chemokine genes in zebrafish can not be attributed to the whole-genome duplication. The phylogenetic and gene organization analyses suggest that extensive intrachromosomal duplication events have occurred in a zebrafish-specific manner after the wholegenome duplication. Particularly, tandem duplications on chromosomes 24 and 25 apparently contributed to the increase in the number of chemokine genes in the zebrafish genome. Catfish might have also increased the different chemokine members by the tandem duplication events in a lineage-specific manner.

Taking advantage of genetic resources such as EST libraries, identification of chemokine genes has been pursued in other fish species such as catfish [28,29,50], trout [31,32], carp [33,35], and cichlid fish [51]. These studies have also shown the existence of species-specific chemokines in each species and the conservation of homeostatic chemokines across species. Each species thus might have acquired and maintained high levels of genetic diversity and the rapid sequence divergence rate in the chemokine superfamily during evolution in order to cope with their respective environmental challenges.

One of the most striking results obtained in this study is the finding of a novel chemokine subfamily CX, which has not been identified in other organisms so far. The genome and gene structures suggest that the generation of this subfamily is the result of the extensive tandem duplication events in the zebrafish genome (Figures 1 and 4). Not only the sequence-structure (Figure 1) but also the *in vitro *chemotactic activity (Figure 6A) showed that it is truly the fifth subfamily of the chemokine superfamily. The highly conserved four cysteine residues in the chemokine molecules are involved in the formation of two disulfide bridges, between the first and the third and between the second and the fourth [52]. Since the CX subfamily lacks one of the two N-terminus cysteine residues but retain the third and fourth cysteine residues together with three additional subfamily-specific cysteine residues, it is interesting to see how they are folded into a three-dimensional structure.

Another interesting result is the observation of the developmentally regulated expression of zebrafish chemokine genes (Figure 5). In particular, CXL-chr24a and CCL-chr11b mRNAs are mainly expressed for a short period of time during the embryonic development but not in adults. These results suggest that each chemokine has highly specific biological functions during the embryonic development other than previously conceived. The involvement of cxcl12a and its receptor cxcr4 in the directed migration of primordial germ cells in zebrafish embryos has been shown [15], and similar observations have been reported in chicken and mouse [53,54]. Therefore, characterization of other chemokine functions in embryos may elucidate the molecular basis of the dynamic movements of various types of cells during vertebrate development. Zebrafish is a model organism suitable for such studies, and our identification and characterization of the zebrafish chemokine genes may be useful for such studies.

## Conclusion

We present the most extensive overall analysis of the chemokine repertoire in zebrafish and compare this to those of other fishes and human. By comparing the chemokine sequences predicted from the zebrafish draft sequence with those of isolated cDNAs and ESTs, the sequences have been manually curated to ascertain a higher level of completeness and quality. Of the species reported previously and two species of pufferfish analyzed here, zebrafish is found to contain the greatest number of chemokine genes comprising CXC, CC and XC subfamilies and a novel subfamily CX. The CX subfamily members lack one of the four cysteine residues highly conserved among the chemokines. One of the CX subfamily members has been proven to be chemotactic for carp leukocytes and to be expressed during a short period time during the embryogenesis, suggesting a role in the developmental processes. Based on the analyses on the genome organization, phylogenetic trees, and synteny, zebrafish chemokines can be divided into zebrafish-specific genes including CX subfamily members, fish-specifi genes, several genes moderately similar to human chemokines, and genes orthologous to human homeostatic chemokines CXCL12 and CXCL14. The fish- or zebrafish-specific chemokines are the most abundant in zebrafish and it is apparent that most of these genes were generated by extensive duplication events only in fish or zebrafish lineages. These chemokines have unique protein structures and gene expression patterns and might be specifically involved in the development and the adult body plans of zebrafish.

## Methods

### Gene prediction

Human chemokine protein sequences were used as queries to search the zebrafish (6th version), fugu (4th version), and *Tetraodon *(7th version) genomic databases at Ensembl [55]. The EST database at the National Center for Biotechnology Information [56] was similarly searched. The searches were repeated using the identified fish chemokine genes and ESTs as queries to identify homologous genes that were hard to be detected by initial searches using the human sequences. Once the fish chemokine gene loci were identified, the surrounding genome sequences were analyzed by exon-predicting program GenScan [57] to detect exons containing amino acid residues conserved among chemokines. Since our predictions of exons and exonintron junctions were based on the comparison with other chemokine genes, mRNAs, ESTs, or proteins, the chemokine sequences described in this study were often different from the NCBI RefSeq provisional records (accession prefix XM_) or the Ensembl genes, which are model mRNAs generated in an automated fashion in the genome annotation process.

### Zebrafish maintenance

Adult zebrafish were maintained in a cycle of 14 h light and 10 h darkness per day in 28.5°C water. Embryos were incubated in 1/3 Ringer's solution (39 mM NaCl, 0.97 mM KCl, 1. 8 mM CaCl2, 1. 7 mM HEPES, pH 7.2) in a 28.5°C water bath. Developmental stages were determined by embryo morphology and hours post fertilization (hpf) [12].

### Isolation of zebrafish chemokine cDNAs

Total RNAs were prepared from various embryonic stages, 0, 4, 8, 12, 24, 36, 48, 70, 120 hpf, and adults (six months) using Trizol reagent (Invitrogen, Carlsbad, CA). The RNAs were reverse-transcribed using oligo-dT. The double-stranded cDNAs were synthesized using PrimeStar HS DNA polymerase (Takara Bio, Kyoto, Japan). These cDNAs were mixed to isolate chemokine cDNAs. The primer sequences were designed based on the genome or EST sequences (Table 2). The PCR condition was 30 cycles of 98°C for 10 s, 60°C for 5 s and 72°C for 1 min. The 5' cDNA fragment of CCL-chrUi was obtained by the RACE (rapid amplification of cDNA ends) method using the Marathon cDNA Amplification kit (Clontech, Palo Alto, CA) as described previously [58]. The gene-specific primer 5'-CCATTTCAGCTGAGGGTCCACACAG was derived from an EST sequence (GenBank accession number BQ615577). The complete cDNA was isolated by RTPCR with the primers in Table 2. To avoid individual differences observed in some isolated cDNA clones, amino acid sequences derived from the draft genome sequences were used for the phylogenetic analyses as far as the genomic sequences of the genes are available. The nucleotide sequences of the cloned cDNAs were deposited in GenBank under successive accession numbers AB331747–AB331780 (see Table 1).

### Computational methods

Signal sequences and O-glycosylation sites were predicted by SignalIP 3.0 [59] and NetOGlyc 3.1 [60], respectively. Transmembrane regions were determined by SOSUI [61]. For phylogenetic analysis, the zebrafish and pufferfish chemokine protein sequences, together with those of human, were aligned with MAFFT [62] and corrected by a visual inspection. For this alignment, sequences corresponding to the chemokine domain (Pfam PF00048 [63]) were extracted. Amino acid distance matrices, JTT (Jones-Taylor-Thornton) or Dayhoff's (PAM), were then used to infer phylogenetic trees by the neighbor-joining method implemented in the MEGA program package version 3 [64]. Statistical support for the clustering was inferred using the interior-branch test [65]. As the number of sequences in the tree increases, the bootstrap test usually underestimates the extent of statistical support of clusters [65]. The interior-branch test is therefore more preferable for the present analysis than the bootstrap test. Human and other sequences used for the tree construction were taken from the NCBI reference and GenBank files, respectively: XCL1, NP_002986; XCL2, NP_003166; CCL1, NP_002972; CCL2, NP_002973; CCL3, NP_002974; CCL3L1, NP_066286; CCL3L3, NP_001001437; CCL4, NP_002975; CCL4L1, NP_001001435; CCL4L2, NP_996890; CCL5, NP_002976; CCL7, NP_006264; CCL8, NP_005614; CCL11, NP_002977; CCL13, NP_005399; CCL14, NP_004157; CCL15, NP_004158; CCL16, NP_004581; CCL17, NP_002978; CCL18, NP_002979; CCL19, NP_006265; CCL20, NP_004582; CCL21, NP_002980; CCL22, NP_002981; CCL23, NP_665905; CCL24, NP_002982; CCL25, NP_005615; CCL26, NP_006063; CCL27, NP_006655; CCL28, NP_683513; CXCL1, NP_001502; CXCL2, NP_002080; CXCL3, NP_002081; CXCL4, NP_002610; CXCL4LV1, NP_002611; CXCL5, NP_002985; CXCL6, NP_002984; CXCL7, NP_002695; CXCL8, NP_000575; CXCL9, NP_002407; CXCL10, NP_001556; CXCL11, NP_005400; CXCL12, NP_000600; CXCL13, NP_006410; CXCL14, NP_004878; CXCL16, NP_071342; CXCL17, NP_940879; CX3CL1, NP_002987. Channel catfish sequences: SCYA3, AF538720; SCYA101, DQ173276; SCYA102, DQ173277; SCYA103, DQ173278; SCYA104, DQ173279; SCYA104v2, AY555513; SCYA105, AY555502; SCYA106, DQ173280; SCYA107, DQ173281; SCYA108, DQ173282; SCYA109, DQ173283; SCYA110, DQ173284; SCYA111, DQ173285; SCYA112, DQ173286; SCYA113, DQ173287; SCYA114, DQ173288; SCYA114B, DQ182570; SCYA115, DQ173289; SCYA116, DQ173290; SCYA117, DQ173291; pSCYA118, DQ173292; SCYA119, DQ173293; SCYA120, DQ173294; SCYA120B, DQ182569; SCYA121, DQ173295; SCYA122, DQ173296; SCYA124, DQ173297; SCYA126, DQ173298; CCL-A, CB939345; CCL-B, CB940829; CCL-C, CB938694; CCL-D, CB938412; CCL-E, CB937843; CCL-F, CB936843; CCL-G, CB937370; CCL-H, CB938775; CCL-I, BM028237; CCL-J, CB937549; CCL-K, CB936954; CCL-L, CV992304; CCL-M, CV992304; IL8, AY145142; CXCL2-like, AY836754; CXCL10-1, AY335949; CXCL10-2, AY335950; CXCL12, AY836755; CXCL14, AY836756; CXCL-A, BE470298; CXCL-B, CK422021; CXCL-C, BE470282; CXCL-D, CF262322; CXCL-E, CV990129. Rainbow trout: CK1, AF093814; CK2, AF418561; CK2.1, AY372431; CK3, AJ315149; CK4A, CA371157; CK5A, BX910748; CK5B, AY561709; CK6, CA355812; CK7A, Omy.33105; CK7B, Omy.9682; CK8A, Omy.16818; CK8B, CA353159; CK9, Omy.16956; CK10, Omy.34323; CK11, Omy.24120; CK12A, CA358073; CK12B, Omy.15592; CCL-A, Omy.12504; CCL-B, Omy.15908; CCL-C, BX909467; IL8, AJ279069; Vig-7, AF483527; CXCd1, DQ191446; CXCd2, DQ191449; CXCL-A, Omy.24155; CXCL-B, BX859166; CXCL-C, Omy.2417; CXCL-D, Omy.23316; CXCL-E, CX035482; CXCL-F, BX308389. Common carp: CK-1, AB010469; CXCa, AJ421443; CXCb, AB082985; CXCL12a, AJ627274; CXCL12b, AJ536027; CXCL14, AJ536028; IL8, DQ453125. European river lamprey: LFCA-1, AJ231072. Shark SCYA107, AB174767.

### Semi-quantitative RT-PCR

RT-PCR was performed using Platinum Taq (Invitrogen). The primers used are listed in Table 3. The amplification conditions, which were carefully chosen to obtain signals in a linear amplification range, were denaturation at 94°C for 30 s, annealing at 60°C for 30 s, and extension at 72°C for 30 s for 32 cycles for the chemokines tested, and 23 cycles for β-actin. Amplification products were electrophoresed on 2% agarose gels.

### Chemokine protein preparation

Zebrafish CXL-chr24a and CCL-chr11a were expressed as a secreted protein containing a (His)_6 _COOH-terminal tag. For this purpose, the chemokine coding sequences were amplified by PCR with the following primers. CXL-chr24a, 5'-TTTGGTACCATGCATCTGTCCTCTGTATCTCA and 5'-TTTTCTAGAAGCCTCCCCTCTACACGTTAC. CCL-chr11a, 5'-TTTGGTACCATGGAAACGCAGAGTTCAACC and 5'-TTTTCTAGACATAGCTCTTTTGTTTTTCTCAT. The PCR condition was as described in the above 'Isolation of zebrafish chemokine cDNAs'. After digestion with *Kpn*I and *Xba*I, the products were ligated into the *Kpn*I/*Xba*I sites of pEF4/Myc-His A vector (Invitrogen). To produce the recombinant proteins, human embryonic kidney HEK293 cells (Invitrogen) were transfected with the expression vectors using Lipofectamine 2000 (Invitrogen). The transfected cells were maintained for 3 days in Dulbecco's modified Eagle's medium supplemented with 10% FCS. The chemokines tagged with (His)_6 _swere purified in a single step with a His-binding Ni-affinity column (QIAGEN, Hilden, Germany) to a purity >95%.

### Chemotaxis assay

Leukocytes of the common carp were prepared essentially as described previously [66]. Cell suspension of the head and body kidneys was layered on an isotonic Percoll (GE Healthcare, Buckinghamshire, England) solution of the specific gravity of 1.090. After centrifugation at 550 g for 40 min, the leukocyte fraction on the Percoll layer was collected and washed with Hanks' balanced salt solution (HBSS) containing 10 mM HEPES, pH 7.3 (HBSS-H). Leukocytes were resuspended in the same buffer and used for chemotaxis assays in a 48-well microchemotaxis chamber (Neuro Probe, Gaithersburg, MD) as described previously [66]. Briefly, the lower compartments of the microchamber were filled with dilutions of chemokine (25 μl) or with control buffer, and the upper compartments with 38 μl of leukocyte suspension (1 × 10^6 ^scells/ml). The two compartments were separated by a 5-μm pore size (PVP-free) polycarbonate filters (Neuro Probe). After incubation at 25°C for 2 h, the filters were removed from the chambers, and the cells that migrated into the lower wells were counted with an improved Neubauer's counting plate (Hirschmann Laborgerate, Eberstadt, Germany). All assays were done in triplicate and the results were expressed as the mean number of cells per 0.4 mm^3^. To determine the cell types attracted by chemokines, cells migrated into the lower wells were analyzed by the May-Grunwald-Giemsa staining.

## Abbreviations

EST: expressed sequence tags; hpf: hours post fertilization; RACE: rapid amplification of cDNA ends

## Authors' contributions

HN planed the entire study and carried out the database searches. KH and ST (Kinki Univ.) performed RT-PCR. NO and JK constructed the phylogenetic trees. YKU and MN carried out the chemotaxis assay. KOO, ST (Kumamoto Univ.), and RM isolated and sequenced the cDNAs. TI, AY and YK prepared the RNAs. HN and OY analyzed the data and wrote the manuscript. All authors read and approved the final manuscript.

## Supplementary Material

Additional file 1 Chemokine genes in pufferfish. Click here for file

Additional file 2 Comaprison of the chemokine genes identified in this study with those reported in other studies. Click here for file

Additional file 3 **Amino acid nucleotide sequences of zebrafish and pufferfish chemokines in FASTA format**. Amino acid nucleotide sequences of zebrafish and pufferfish chemokines in FASTA format. To avoid individual differences observed in some isolated cDNA clones, amino acid sequences shown in this figure are derived from draft genome sequences as long as the genomic sequences for the genes are available. Small letters indicate signal sequences. Red letters show the conserved cysteine residues and the WV (tryptophan and valine) motif, and green letters the three cysteine residues observed in the CX subfamily and some CC members. The amino acid residues at the splicing sites and the transmembrane regions are highlighted in grey and green, respectively. Abbreviations: z, zebrafish; f, fugu; t, *Tetraodon*. Click here for file

Additional file 4 **A phylogenetic tree constructed using all chemokine subfamily members**. The tree was constructed using the Dayhoff matrix and the neighbor-joining method. Numbers at branch nodes represent the confidence of bootstrap test with 1000 iterations. Confidence values of ≥30% are shown. The symbols used are the same with those in Figure 2. Click here for file

Additional file 5 **Pairwise comparison (*p*-distances) of the zebrafish chemokine genes**. Identities on the amino acid level were calculated using the MEGA program package version 3. Red and yellow cells indicate identical or more than 90% similar gene pairs, respectively. Click here for file
