# Supplementary material for: Extensive expansion and diversification of the chemokine gene family in zebrafish: Identification of a novel chemokine subfamily CX
Source: BMC Genomics. 2008 May 15;9:222. doi: 10.1186/1471-2164-9-222 (PMC2416438; doi:10.1186/1471-2164-9-222)
Supplement: Additional file 1 — Chemokine genes in pufferfish. [file 1471-2164-9-222-S1.doc]

**Additional file 1**

**Chemokine genes in pufferfish***^a^*

|  | **Tetraodon** | | | | | | |  | **Fugu** | | | | |
| --- | --- | --- | --- | --- | --- | --- | --- | --- | --- | --- | --- | --- | --- |
| **Chemokine** | **Chr** | **Location** | **Orienta-**  **tion^b^** | **Exons^c^** | **cDNA** | **Length (a.a.^d^)** | **O-Glycosyla-**  **tion^e^** |  | **Scaffold** | **Location** | **Orienta-**  **tion** | **Exons^c^** | **EST/**  **UniGene^f^** |
| **CC** |  |  |  |  |  |  |  |  |  |  |  |  |  |
| CCL-chr18a | 18 | 7004875 - 7006286 | + | 3 | CR734658 | 91 | 0 (0) |  |  |  |  |  |  |
| CCL-chr15a | 15 | 2593994 - 2594863 | + | 4 | CR660778 | 105 | 0 (0) |  | 90 | >451800 - 452441 | – | (4)^g^ | Tru.2876 |
| CCL-chr15b | 15 | 4408647 - 4410403 | – | 4 |  | 105 | 0 (0) |  | 214 | 422978 - 423786 | – | 4 |  |
| CCL-chr15c | 15 | 5048784 - 5049194 | + | 3 |  | 82 | 1 (1) |  | 73 | 1034531 - 1034954 | – | 3 |  |
| CCL-chr13a | 13 | 826023 - 826850 | + | 3 |  | 94 | 0 (0) |  | 241 | 206929 - 207441 | + | 3 |  |
| CCL-chr12a | 12 | 3595838 - 3596442 | + | 3 | CR734478 | 99 | 0 (0) |  | 4 | 1478547 - 1479037 | – | 4 | AL838273 |
| CCL-chr12b | 12 | 3597842 - 3598454 | + | 4 |  | 99 | 0 (0) |  | 4 | 1482196 - 1482801 | + | 4 |  |
| CCL-chr12c | 12 | 3601359 - 3602432 | + | 4 | CR719468 | 116 | 0 (0) |  | 4 | 1485571 - 1486601 | + | 4 | CA330300 |
| fCL1 | 12 | 9151252 - 9151739 | – | 3 | CR666818 | 94 | 0 (0) |  | 11 | 2288356 - 2289066 | – | 3 |  |
| CCL-chr1a | 1 | 10663714 - 10664550 | – | 3 | CR660806 | 99 | 0 (0) |  | 135 | 288528 - 289474 | – | 3 | AL836165 |
| CCL-chrUa | Unk |  |  |  | CR632585 | 95 | 0 (0) |  |  |  |  |  |  |
| CCL-chrUb |  |  |  |  |  |  |  |  | 73 | 1036349 - 1036825 | + | 3 |  |
| CCL-chrUc |  |  |  |  |  |  |  |  | 135 | 306939 - 308554 | + | 3 |  |
| **CXC** |  |  |  |  |  |  |  |  |  |  |  |  |  |
| **il8**^h^ | 18 | 150729 - 153192 | + | 4 |  | 116 | 0 (0) |  | 333 | 210249 - 210851 | + | 4 | Tru.2042 |
| CXCL-chr17a | 17 | 4657739 - 4658498 | + | 4 |  | 136 | 5 (6) |  | 24 | 839530 - 840362 | – | 4 | CA844719 |
| CXCL-chr17b | 17 | 4681725 - 4685886 | + | 4 | CR726361 | 98 | 0 (0) |  | 24 | 810353 - 814682 | – | 4 |  |
| CXCL-chr12a | 12 | 9986492 - 9987158 | – | 4 |  | 102 | 1 (1) |  |  |  |  |  |  |
| CXCL-chrUa | Unk | 21693066 - 21693950 | + | 3 |  | 105 | 1 (1) |  | 421 | 105821 - 106809 | + | 3 |  |
| CXCL-chrUb | Unk | 90685815 - 90689346 | – | 4 |  | 90 | 0 (0) |  | 5 | 2459360 - 2463125 | – | 4 | BU808394 |
| CXCL-chrUc | Unk | 129480789 - 129481455 | – | 4 | CR721508 | 89 | 0 (0) |  | 7 | >361001 - 361602 | – | (4)^g^ |  |
| CXCL-chrUd |  |  |  |  |  |  |  |  | 421 | 102340 - 1027526 | + | 3 |  |

^a^ Version 4 (fugu) and version 7 (tetraodon) assemblies were used for the analyses (http://www.fugu-sg.org/project/info.html).

^b^ Relative transcriptional orientation.

^c^ Number of exons containing coding sequences.

^d^ Amino acid residues.

^e^ Number of O-glycosylation sites in the COOH-terminal region and in the whole mature peptide (parentheses).

^f^ Tru numbers are Unigene IDs (Build #3, http://www.ncbi.nlm.nih.gov/sites/entrez?db=unigene).

^g^ The last exon sequences have not yet been determined.

^h^ Bold chemokine names are approved gene symbols.
