# Supplementary material for: Extensive expansion and diversification of the chemokine gene family in zebrafish: Identification of a novel chemokine subfamily CX
Source: BMC Genomics. 2008 May 15;9:222. doi: 10.1186/1471-2164-9-222 (PMC2416438; doi:10.1186/1471-2164-9-222)
Supplement: Additional file 2 — Comaprison of the chemokine genes identified in this study with those reported in other studies. [file 1471-2164-9-222-S2.doc]

**Additional file 2**

**Comaprison of the chemokine genes identified in this study with those reported in other studies**

| **our study** | **DeVries et al (2005)** | **Peatman and Liu (2006)** |
| --- | --- | --- |
| **zebrafish** |  |  |
| CCL-chr25a | dr-chr25-CCL8-30.8%-EP41974a | (Dr25_WGA1873_1_709505) ^a^ |
| CCL-chr25b | (dr-chr25-CCL8-35.6%-EP41974b) | - |
| CCL-chr25c | - | - |
| CCL-chr25d | - | - |
| CCL-chr25e | - | - |
| CCL-chr25f | - | - |
| CCL-chr25g | - | (Dr25_WGA1872_1_17999) |
| CCL-chr25h | - | - |
| CCL-chr25i | - | - |
| CCL-chr25j | - | - |
| CCL-chr25k | - | (Dr25_WGA1873_1_857912) |
| CCL-chr25l | - | - |
| CCL-chr25m | - | - |
| CCL-chr25n | - | - |
| CCL-chr25o | - | - |
| CCL-chr25p | - | - |
| CCL-chr25q | - | - |
| CCL-chr25r | - | - |
| CCL-chr25s | - | - |
| CCL-chr25t | (dr-chr25-CCL13-38.9%-DN) | - |
| CCL-chr25u | (dr-chr25-CCL2-37.5%-DN) | - |
| CCL-chr25v | (dr-chr25-CCL13-35.9%-DN) | - |
| CCL-chr25w | (dr-chr25-CCL11-31.2%-DN) | (Dr25_WGA1872_1_141073) |
| CCL-chr25x | (dr-chr25-CCL2-36.4%-DN) (dr-chr25-CCL2-35.0%-DN) | - |
| CCL-chr25y | (dr-chr1-CCL26-33.3%-EP26489) | (XP_696742_Dr1_WGA6_1_97332) |
| CCL-chr25z | - | - |
| CCL-chr25aa | - | - |
| CCL-chr25ab | - | (XP_696742_DrUn_WGA2406_1_18581) |
| CCL-chr25ac | - | (XP_696742_Dr25_WGA1872_1_19737) |
| CCL-chr24a | dr-chr24-CCL20-34.6%-DN | CO802793_Dr24_WGA1806_1_416955 |
| CXCL-chr24a | - |  |
| CXCL-chr24b | - |  |
| CXCL-chr24c | - |  |
| CXL-chr24a | - | - |
| CXL-chr24b | - | - |
| CCL-chr24b | - | - |
| CCL-chr24c | - | - |
| CCL-chr24d | - | - |
| CCL-chr24e | - | - |
| CCL-chr24f | - | - |
| CXCL-chr24d | - |  |
| CXCL-chr24e | - |  |
| CCL-chr24g | - | - |
| CCL-chr24h | (dr-scNA5340-CCL19-30.5%-DN) | - |
| CCL-chr24i | dr-chr24-CCL19-33.3%-EK41769 | - |
| CCL-chr24j | - | (DN898053_DrUn_WGA7827_1_17321) |
| CCL-chr24k | - | (DrUn_WGA7827_1_9830) |
| CCL-chr24l | (dr-NA5340-CCL3-31.3%-DN) | (DrUn_WGA7827_1_7256) |
| CCL-chr24m | dr-chr22-CCL19-30.3%-DN | AW233060_Dr22_WGA1631_1_283786 |
| CCL-chr24n | - | - |
| CCL-chr23a | - | - |
| cxcl12bL | drCXCL12c |  |
| **cxcl12b**^f^ | drCXCL12b |  |
| CCL-chr20a | - | - |
| CCL-chr20b | - | (CN504135_Dr20_WGA1544_1_380308) |
| CCL-chr20c | (dr-chr20-CCL24-35.6%-DN) | (BM183274_Dr20_WGA1544_1_382837) |
| CCL-chr20d | - | (AL920330_Dr20_WGA1544_1_386533) |
| CCL-chr20e | (dr-chr20-CCL14-32.9%-DN) | (Dr20_WGA1544_1_390316) |
| CCL-chr20f | (dr-chr20-CCL26-33.3%-DN) | (Dr20_WGA1544_1_375272) (Dr15_WGA1175_1_212813) ( Dr20_WGA1544_1_401369) |
| CCL-chr20g | (dr-chr20-CCL7-40.0%-DN) | (CAI20929_Dr20_WGA1544_1_144682) |
| CXL-chr19a | - | - |
| CCL-chr17a | - | - |
| CCL-chr17b | - | - |
| CCL-chr17c | - | - |
| **scyba**^f^ | drCXCL14a, (drCXCL14b) |  |
| CXCL-chr13a | - |  |
| CXCL-chr13b | dr-chr13-CXCL9-28.0%-EK14232 |  |
| **cxcl12a**^f^ | z-cxcl12a |  |
| CXCL-chr13c | - |  |
| CXCL-chr13d | (dr-scNA11550-CXCL2-36.0%-DN) |  |
| CXL-chr12a | - | - |
| CCL-chr11a | (dr-chr11-CCL21-36.7%-DN) | (CO921699_Dr11_WGA839_1_159228) |
| CCL-chr11b | - | BI475311_Dr11_WGA839_1_233313 |
| CCL-chr10a | - | CO916907_Dr10_WGA780_1_413112 |
| CCL-chr10b | (dr-chr9-CCL17-22.3%-DN) (dr-chr9-CCL13-28.2%-DN) | (BI839410_Dr9_WGA697_1_854420) |
| **ccl1**^f^ | (dr-chr18-CCL11-34.7%-DN) | AAF17560_Dr8_WGA606_1_66363 AAF17560_Dr18_WGA1436_1_236332 |
| CCL-chr7a | (dr-chr7-CCL13-32.5%-DN) | - |
| CCL-chr5a | (dr-scNA10579-CCL19-34.3%-DN) | (CO360769_DrUn_WGA13047_1_39000) |
| CCL-chr5b | - | (Dr9_WGA710_1_710182) |
| CXCL-chr5a | dr-chr5-CXCL8-31.2%-EK2963 |  |
| CXCL-chr5b | (dr-chr5-CXCL11-25.2%-EP22087) |  |
| CXCL-chr5c | (dr-chr5-CXCL10-28.6%-DN) |  |
| CXCL-chr5d | (dr-chr5-CXCL11-37.9%-EP13940) |  |
| CXCL-chr5e | (dr-chr5-CXCL11-37.2%-EK12810) |  |
| CXCL-chr5f | (dr-chr5-CXCL9-35.1%-EK43819) |  |
| CXCL-chr5g | (dr-chr1-CXCL11-37.1%-DN) |  |
| CXCL-chr5h | - |  |
| CXCL-chr5i | dr-chr1-CXCL9-37.2%-EK42273 |  |
| CCL-chr2a | (dr-scNA3668-CCL20-31.6%-DN) (dr-scNA3668-CCL14-26.8%-EK2252) | (DrUn_WGA6170_1_12898) |
| CCL-chr2b | (dr-chr20-CCL11-29.1%-EP37660) | - |
| CCL-chr2c | - | (BM533896_Dr7_WGA489_1_245599) |
| XCL-chr2a | - | - |
| CCL-chr2d | (dr-chr11-CCL19-32.7%-DN) (dr-chr11-CCL19-33.7%-DN) | - |
| CCL-chr2e | - | CO913970_Dr11_WGA879_1_755946 CO913970_Dr11_WGA879_1_759152 CO913970_Dr11_WGA879_1_762108 |
| CCL-chr2f | (dr-chr11-CCL20-32.3%-EK35666) | - |
| CXCL-chr1a | - |  |
| CXCL-chr1b | (dr-chr1-CXCL8-35.4%-EK34299) |  |
| CXCL-chr1c | dr-chr25-CXCL11-28.0%-EP27297 |  |
| CCL-chr1a | - | (DrUn_WGA6170_1_11888) |
| CCL-chr1b | - | (Dr20_WGA1527_1_1613261) |
| CCL-chr1c | - | - |
| CCL-chrUa | - | - |
| CCL-chrUb | - | - |
| CCL-chrUc | - | - |
| CCL-chrUd | - | - |
| CCL-chrUe | - | - |
| CCL-chrUf | - | - |
| CCL-chrUg | - | CK237158_Dr20_WGA1527_1598576 |
| CCL-chrUh | - | - |
| CCL-chrUi | - | - |
| CCL-chrUj | (dr-chr25-CCL3-27.4%-DN) | (CN326771_Dr25_WGA1872_1_176060) |
| **pufferfish** |  |  |
| tCCL-chr18a | - |  |
| **f-il8** | (fr-sc30-CXCL8-36.5%-EK149334) |  |
| fCXCL-chr17a | fr-sc28-CXCL5-27.6%-DN |  |
| fCXCL-chr17b | - |  |
| fCCL-chr15a | - |  |
| fCCL-chr15b | (fr-sc206-CCL20-37.0%-DN) |  |
| fCCL-chr15c | (fr-sc509-CCL5-34.8%-DN) |  |
| fCCL-chr13a | - |  |
| fCCL-chr12a | fr-sc687-CCL3-31.2%-EK171148 |  |
| fCCL-chr12b | (fr-sc687-CCL3-31.2%-EK177216) |  |
| fCCL-chr12c | (fr-sc687-CCL18-27.4%-EK168760) |  |
| fCL1 | (fr-sc1096-CCL28-28.9%-EK174502) |  |
| tCXCL-chr12a | - |  |
| fCCL-chr1a | (fr-sc114-CCL21-33.3%-EK167779) |  |
| tCCL-chrUa | - |  |
| fCCL-chrUb | (fr-sc509-CCL17-35.6%-DN) |  |
| fCCL-chrUc | - |  |
| fCXCL-chrUa | - |  |
| fCXCL-chrUb | frCXCL14 |  |
| fCXCL-chrUc | - |  |
| fCXCL-chrUd | (fr-sc605-CXCL11-30.4%-EK168958) |  |

^a^ Similar but not identical to our sequences are shown in parentheses.
