# Supplementary material for: Extensive expansion and diversification of the chemokine gene family in zebrafish: Identification of a novel chemokine subfamily CX
Source: BMC Genomics. 2008 May 15;9:222. doi: 10.1186/1471-2164-9-222 (PMC2416438; doi:10.1186/1471-2164-9-222)
Supplement: Additional file 3 — Amino acid nucleotide sequences of zebrafish and pufferfish chemokines in FASTA format. Amino acid nucleotide sequences of zebrafish and pufferfish chemokines in FASTA format. To avoid individual differences observed in some isolated cDNA clones, amino acid sequences shown in this figure are derived from draft genome sequences as long as the genomic sequences for the genes are available. Small letters indicate signal sequences. Red letters show the conserved cysteine residues and the WV (tryptophan and valine) motif, and green letters the three cysteine residues observed in the CX subfamily and some CC members. The amino acid residues at the splicing sites and the transmembrane regions are highlighted in grey and green, respectively. Abbreviations: z, zebrafish; f, fugu; t, Tetraodon. [file 1471-2164-9-222-S3.doc]

### Additional file 3

### Amino acid nucleotide sequences of zebrafish and pufferfish chemokines in FASTA format

>zCCL-chr25a

mrassvflllgltvlmawtseaQPAIPEPCCFNFIDFPIPANKVVSAVRTGSRCAVKGIVVTTPRTQFCVKPDEDWIKPIMEKQQ

>zCCL-chr25b

mssppcpwmsttttlapattaITTRAKTTPLPKPKQQIDRSSIPGPAAVLITCCFAFIDFTIPYNKIVSALRTSPRCAAKAIVVTTPRTQFCVKPNELWIKAVMEKQLQK

>zCCL-chr25c

mkiimktallfavlccallpqpsdgQESADAANSMCCFGKGSNIKIPLRRLEYFYWTSSRCPLKHVVFVTIAKKHLCMNPDNEWVQKVINMKSGSGSSV

>zCCL-chr25d

mktpllllvcvvmlcslpdsssgQESIDAGNSICCFGNSNSRIPLKRLKYFYWTSSHCPFKHIVFVTIAKRHICMNLDNEWVQKVISMKSVSGSPV

>zCCL-chr25e

mktpllllvcvvmlyslpdsssgQQSIRKGICCFGKGSNRRIKLNRLNSYYWTSNFCTLKRLVFVTTTKRNICMNPENEWVQKIIKEKLILDLSI

>zCCL-chr25f

mkiimktallfavlccallpqssdgQPYSGDASTSICCFGKGSNSKIPPNRLKSYYWTSSICPFNHIVFVTAKRHICMNPENKWVQKTMKAIDKKPGSNSPV

>zCCL-chr25g

mrassvflllgltvlmawtseaQPAIPEPCCFNFIDFPIPANKVVSAVRTVSRCAVKGIVVTTPRTQFCVKPDEDWIKPIMEKQQ

>zCCL-chr25h

mssppcpwmsttttlapattaITTRAKTTTLPKPKRQIDRSSIPGPAAVPITCCFAFIDFPIPYNKIVSALRTSPRCATKAIVKTPKTQFCVKPDKDWIRPIMERKLQK

>zCCL-chr25i

mkiimktallfavlccallpqssdgQPYSGDASTSICCFGKGSNSKIPPNRLKSYYWTSSICPFNHIVFVTAKRHICMNPENKWVQKTMKAIDKKPGSNSPV

>zCCL-chr25j

mktpllllvcvvmlyslpdsssgQQSIRKGICCFGKGSNRRIKLNRLNSYYWTSNFCTLKRLVFVTTTKRNICMNPENEWVQKIIKEKLILDLSI

>zCCL-chr25k

mktpllllvcvvmlcslpdsssgQESIDAGNSICCFGNSNSRIPLKRLKYFYWTSSHCPFKHIVFVTIAKRHICMNLDNEWVQKVISMKSVSGSPV

>zCCL-chr25l

mkiimktallfavlccallpqpsdgQESADAANSMCCFGKGSNIKIPLRRLEYFYWTSSRCPLKHVVFVTIAKKHLCMNPDNEWVQKVINMKSGSGSSV

>zCCL-chr25m

mrslmfllvlvlfccvqetscAPLAMNSAKSVCCEATTDKNIPLKQIMSYQWTTSTCPIKAIVFKTIAGRKICVDPQNTLVKNQVAKLDKRTSSTTALSPESTSTTAETPAATSAAASSSEFTSATSSQASTSATALSHESTSATSSPESTSVSKSSPESTSAKSSPASTSATALSPEYTSASTVTFTTESHSTSDQSAVI

>zCCL-chr25n

mrsllfllvlmlfcylqaTSSSMEAINSEKSVCCEGFTHKKIPLKQIVSYLWTSSNCAVKVIVYDKSRKKICVHPENNFVKRQVVILDSRAKV

>zCCL-chr25o

mrslmfllvlvlfcslqdtssaMDAIISANSVCCEGITHKKITLKQIVSYHWTTSSCAKKAIVFTTKAGKKICVDPENTFVKRQVVILDSRAKV

>zCCL-chr25p

mrslmfllvlvlfccvqetscSPIPINSAKSVCCEATTHRNIPLKQIMSYQWTTSMSYKSHCVGTIAGREICVDPQNTLVKKQVAKLDKRRSTTALSPKSTSTTAETPAATSGTTSSSESTSAKSSPASTSATALSHEYTSASTVTFTTAESHSTSV

>zCCL-chr25q

mrslmfllvlvlfcslqdtssaMEATISANSVCCEGFTHKKIPLSKIVSHHLTTSNCAKKFIVFTTKAGKKICVDPENTFVKRQVAELDSRTRV

>zCCL-chr25r

mripvfllflvftmcsiqlvpaMPAIPEFCCINFIDFPIPANKIVSAVITPSRCSSKGIMVTTPRTQFCVKPDEDWIKPIMEKQYKR

>zCCL-chr25s

mripvfllflvftmcsiqlvpaYPVPESCCFNFIDFPIPANKIVSAVRTSSRCSSKGIMVTTPRTQFCVKPDEDWIKPIMEKQ

>zCCL-chr25t

mrslmfllvlvlfcslqdtssaMEAIISANSVCCEGFTHKKIPLSKIVSYHLTTSNCAKKFIVFTTKAGKKICVDPENTFVKRQVAELDSRTRV

>zCCL-chr25u

mrslmfllvlvlfccvqetscSPIPINSAKSVCCEATTHRNIPLKQIMSYQWTTSTCPIKAIVFKTIAGREICVDPQNTLVKKQVAKLDKRRSTTALSPKSTSTTAETPAATSGTTSSSESTSAKSSPASTSATALSHEYTSASTVTFTTAESHSTSV

>zCCL-chr25v

mrslmfllvlvlfcslqdtssaMDAIISANSVCCEGITHKKITLKQIVSYHWTTSSCAKKDIVFTTKAGKKICVDPENTFVKRQVVILDSRVKV

>zCCL-chr25w

mrsllfllvlmlfcylqaTSSSMEAINSEKSVCCEGFTHKKIPLKQIVSYLWTSSNCAVKVIVYDKSRKKICVHPENNFVKRQVVILDSRAKV

>zCCL-chr25x

mrslmfllvlvlfccvqetscAPISINSAKSVCCKGITHINIPLKQIMSYQWTTSTCPIKAIVFKTIAGREICVDPQNTLVNEQVAKLDKRRSSTTALSPESTSTIAETPAATSATASSSEFTSATSSQASTSATASSSEFTSATSSQASTSATASSSEFTSATSSQASTSATASSSEFTSATSSQASTSATASSSEFTSATSSQASTSATALSHESTSAISSPESTSVSKSSPESTSPKSPPASTSATALSPEYTSASTVTFTTESHSTSDQSAVI

>zCCL-chr25y

mstsrfvflsavvvllcavslsqgMRIGPKRCCFQYAERQVPFKQVMEYSMTSQQCPKEAVLFKTARGRYVCARPSDPWVQEYMQAIDSKRVGNQGTL

>zCCL-chr25z

mripvfllflvftmcsiqlvpaMPATPIAINSAKSVCCEGITHKVIPLKQIMSYQWTTSTCPIKAIVFKTIAGREICVDPQNTLVKKHIANLDKRTSSTTALSPESTFTTAETPASTSATASSSESTSAKSSPASTSATALSHEYTSASTVTFTTAESHSTSV

>zCCL-chr25aa

mripvfllflvftmcsiqlvpaMPATPIAINSAKSVCCEGITHKVIPLKQIMSYQWTTSTCPIKAIVFKTIAGREICVDPQNTLVKKHIANLDKRTSSTTALSPESTFTTAETPASTSATASSSESTSAKSSPASTSATALSHEYTSASTVTFTTAESHSTSV

>zCCL-chr25ab

mstsrfvflsavvvllcavslsqgMRIGPKRCCFQYAERQVPFKQVMEYSMTSQQCPKEAVLFKTARGRYVCARPSDPWVQEYMQAIDSKRVGSQGTL

>zCCL-chr25ac

mstsrfvflsavvvllcavslsqgMRIGPKRCCFQYAERQVPFKQVMEYSMTSQQCPKEAVLFKTARGRYVCARPSDPWVQEYMQAIDSKRVGSQGTL

>zCCL-chr24a

mgnikictlyfivllsflvetesAICCLRYVKNPRRCGFLKGYDIQIMTEGCDLPAIIFHTVTGRSICANPSQNWTQERVLCLKKKAETMKTKTMSMFTTLS

>zCXCL-chr24a

mkcvlitlficlttmllcKDSYQKRSSSYCPCLKTSDIVLRKANIKSYTRQRADVCYIDAIVFKTVKGKTFCADPKKTWVKDAMESLDKKKAAPGIKTTAQPIGSTLNTASTLSTTEDSAENGLDELQTELNEYSTSLFCFFFLFIVFSVFSPMHHIGIERTSLLFVCVSAATGRSFCPCLKTAEVV

>zCXCL-chr24b

mkcvlitlfvcltimllckdsyqttgRSFCPCLKTAEVVMNKEDIRSYKIHNADVCHVDTVEFKTVKGKTFCADPKKTWVKDAMESLDKKKAAPGIKTTAQPIGSTLNTASTLSTTEDSAENESDELQTELNEYSTSLYFFSVHCLFPNTVII..

>zCXCL-chr24c

mrsalitllclavmllvqesyqvsssSYCPCLKLSDGVLRKANIKSYIRQTAGVCHIDAIVFTTVRGITFCADPKLTWVIDAMKFLDKKKAASEPKTTTQPINSTFNATSMPNTTANLNTTNTTSDLNTTNTNTTSHLNTTNTNTKAQTKRLFTTIQPC

>zCXL-chr24a

mhlssvshqmvisshlllllyaftsvvfisvsegWSSTDKNFDNRPGVCFKVLTTKEPKANIKR**C**YNLPKTNNCLK**C**VLFVDASNRMKCIDPNASWLAERLYRLKEKGVT**C**RGEA

>zCXL-chr24b

mdpssasyqmiilsclflllsaFTPDDSHISQNNGLQAKKAVDQKGKICFRNPTTEEPQIEIRA**C**FNLRKTRHCPA**C**VLFIDKKNRMCCINLKAPWLSAKIEHLEKKGIK**C**KNKH

>zCCL-chr24b

mhlstashqliissalllllcasasgDYFMPVMSPVTPPESAPMDLRGMVCCKKFTRKEPQIKINS**C**FFVQEISNCLKSVVLIDEMNKMHCIHPKAPWLNARIKRLEEIGVQ**C**TVH

>zCCL-chr24c

mrlssafhqmiilsallfllcaftsgDHLLQVAYPVPTRDSRFDRKDTVCCRTLTTNEPQIKINS**C**YFLQEISKCLKSVLFIDVKNKMHCIDPTAPWLEARIKRLEENGVK**C**IKTKAH

>zCCL-chr24d

mrlfsasyqmiissalllllcaftsgDHLLQVAHPVLTQDATDSDPKEPVCCKTLTTNEPQIKINSCYFLQETSDCLK**C**VLFVDDMNRMYCIDLTAPWLSERIKCLEEDGVQ**C**INKPNNAYIGTGHL

>zCCL-chr24e

mrlssashqlillsclllllsaftsgGLVTMFIFCHYCYNLISHDSKPKTCCRKYEKNAYFKIQR**C**YILPETDKCLNSIVFTDPNNRNHCYDPTAPWMTIRMNLLKKNNIP**C**KDYTKS

>zCCL-chr24f

mrlssashqlillsclllllsavtsRVMKNLKEMYLTATYMAFHNPKPSTCCEKHETNLPKIQLKK**C**LILPATDKCLESVLFVDIRNRRHCFSTTAPWINERIAIFEKKHGK**C**EKKY

>zCXCL-chr24d

mkcvlitlficlttmllcKDSYQKRSSSYCPCLKTSDIVLRKANIKSYTRQRADVCYIDAIVFKTVKGKTFCADPKKTWVKDAMESLDKKKAAPGIKTTAQPIGSTLNTASTLSTTEDSAENGLDELQTELNEYSTSLFCFFFLFIVFSVFSPMHHIGIERTSLLFVCVSAATGRSFCPCLKTAEVVMNEEDIKSYKIHNADVCHVDTVE

>zCXCL-chr24e

mrsalitllclavmllvqesyqvsssSNCPCLKLSDGVLRKANIKSYIRQRAGVCHIDAIVFTTVRGITFCADPKLTWVIDAMKFLDKKKAASEPKTTTQPINSTFNATSMPNTTANLNTTNTTSDLNTTNTNTTSHLNTTNTNTKAQTKRLFTTIQPC

>zCCL-chr24g

msstigfpfclvlvllcynaatsVRLNCCLRTSKSSIPIKRVVDYRVQQPGICPIEAVILVTVKGKRICCDPNTEWIKKTMRKVDQKKLRKQNSDLKASPNPNNNINQRKRRRQN

>zCCL-chr24h

mdkrilmrsfaivvivsaiwtvtadaERVINCCKSVSTVKVTDPIIGVRIQRKSLPCVNAIIFETDRGHFCSDPRQPWVQRKAQQFIRNLKISQQTSTSLPTSSISERNVEGSAKAPDRL

>zCCL-chr24i

metqrilvrsltivviasaiwpvtdaiEEKGTNCCKSVSTVEVTDPIIGVRMQHQSLPCVNAIIFETDRGDFCSDPRQPWVRRKVKQFIRNFKTNQQTSTQTLTSTPTLTSTPPLTFTPEPTSSISEQTNGEGYA

>zCCL-chr24j

mawtarlltiavlialmgcftgaqaNYRRPTRVGVTCCKEVSRGRIPPDIKLTAYKHQPALSPCVDAIIFYAEKERYCTDPKARWIQNRLQGLKELND

>zCCL-chr24k

metqkilvlnwavvliasviwcittdaKDMMLCCTSVSRLEVTDPIIGFRIQRESRPCVNAILFETERGAFCSSPRQPWVRSKVMQFLAQRNSPTSPLPPLSSATSNE

>zCCL-chr24l

mskdshlklgccaiasviwcitidaKTVMPCCTSVSTAEVTDPIISVGIQRESPPCGTPIIFETKEGKICSDPRHEWVLRKVVQFLTQINSPTSPLPPLSSTTSNE

>zCCL-chr24m

mktqkifmrslavaltasviwtvtavaDNVESCCTPVSTPELTDPIMSVRIQFESLECETAIVFKTEERELCSDPRQLWVRRKVMQFYKNKVTKKTN

>zCCL-chr24n

mrlssashqlillsclllllsavtsRFMKNLKEMYITATYMAFHNPKPSTCCEKHETNLPKIQLKK**C**LILPATDKCLESVLFVDIRNRRHCFSTTAPWINERIAIFEKKHGK**C**EKRY

>zCCL-chr23a

mkfnqfaaflfsiqwmifgneqqvygNEHPFLCCVTVKNIRIPACNIERYSIQKPPLCPIKAVRFHTKKNKVICSDPNSDWAKKVIQQLSQTPALKTALQCYTKSVQTTTATQKIPGTHSETSTTVQPTVTIIHTFEPETSSKINTIGPETSTSTTLKTIETSRAEYSQKTTIATSGLMEDRKTSTETTETDQQDPLSKVQEKQVTSLATSVKLLSKVDDAHKNTNNYFCLKKEKKAIYFCIKQT

>z-cxcl12bL

mdskvvalvallmlafwspetdaKPISLVERCWCRSTLNTVPQRSIREIKFLHTPSCPFQVIAKLKNNREVCINPKTKWLQQYLKNALNKIKKKRSE

>z-**cxcl12b**

mdskvvalvallmlafwspetdaKPISLVERCWCRSTLNTVPQRSIREIKFLHTPSCPFQVIAKLKNNREVCINPKTKWLQQYLKNALNKIKKKRSE

>zCCL-chr20a

mrtscifiaslvlvafcamarsEWSQSPDKCCFSFSNARIPVKQVVSYHTTHLQCNMNGVIFITRAQKEICTNPTEKWVQRLMKMVDNQNMKQMTEAGSADSP

>zCCL-chr20b

mrtscifiaslvlvafcamarsEWSQSPDKCCFSFSNARIPVKQVVSYHTTHLQCNMNGVIFITRAQKEICTNPTEKWVQRLMKMVDNQNMKQMTEAGSADSP

>zCCL-chr20c

mrpscisivclvlfafcsvdgSDLSQSPDKCCFSFSNTRIPVKQVESYHTTHLLCSGNGVIFITKAQREICTNPTEKWVQRLMKLVDNQNMKQMTEAGSGDSA

>zCCL-chr20d

mrqscifiaclvlvafcsvdgSDFSQNPDKCCFSFSTIKIPVKQVQSYHTTHFQCQKNGIIFVTEQKEICADPTERWVQRLMNLVDARLVKDTEASSNGSP

>zCCL-chr20e

mkpscnfiaclvlatfclvnggwSQGPVKCCYSFFNARIPVKEVGGYHATHLQCNINAVIFITKAQREICTNPAEKWVQRLMRLVDVQNMKQMTEGRIGDSLDTHRSKPLHEMPKTNQAVSKKAEIITMTEMPLQQQDETTSILYYFQDWTDFDTTQANSKTESVYFCVRPGGE

>zCCL-chr20f

mrsfcifiillalvalcsaVSQIECCFSFSTVRIPVNQVQSYQTTHFECHKKGIVFITKIQKEICTDPTEEWVQRLMGLVDARYILQTTKSGLVDTHRSKPLHEMPETIPETDAMAK

TAALIKTTDMPLDETTLTSILKESQDWRDVDTTQENSKTQSAYFCM

>zCCL-chr20g

mrrscifiaslvlvafcsvvgSDWSQWSQGPEKCCFSFTNARIPLKQIESYYTTHLQCNMNAVIFIIRAQREICTNPTEKWVRRLMKMVDNQNMKQMTEAGSVDSA

>zCXL-chr19a

mrlssashqmiissglllllcvftsgvFISVSDRIQGRSSSTKNYDARPKLCFQVLTTEQPKANITS**C**YNLPKTSNCLE**C**VLFVDATNRMMCIDPNASWLSERLNRLEAKGVT**C**V

>zCCL-chr17a

mmsicwklalmmmvvlvtsQKASIFTDATDKYGTNCCKSVSKVEVTDPIIGIRIQRKSHLCVKAIIFETEQGDFCSDPRQPWARRKVQQFLRTLFIKTISTPPPTSSISEM

>zCCL-chr17b

mrlfsasyqmiissalllllcaftsgDHLLQVAHPVLTQDATDSDPKEPVCCKTLTTNEPQIKINS**C**YFLQETSDCLK**C**VLFVDDMNRMYCIDLTAPWLSERIKRLEEDGVQ**C**INKPNNAHTGTGHL

>zCCL-17c

mrlssafhqmiilsallfllcaftsgDHLLQVAYPVPTRDSSRFDRKDTVCCKTSTTNEPQIKINS**C**YFLQEISKCLKSVLFIDVKNKMHCIDPTAPWLEARIKRLEE..

>z-**scyba**

mnrcstaalfllviaiyslnteaYKCRCTRKGPKIRYIDVQKLEIKPKHPYCQEKMIFVTMENVSRFKGQEYCLHPRLQSTRNLVKWFKIWKDKHRTFEA

>zCXCL-chr13a

mtqiaytllalnlcfiltaqvvesQHVPKTCQCPQVQKRVRGPFSDLRITPKGPSCLQNEIIVTPKKTNKPVCLSPEGPQGKSLMKCWNR

>zCXCL-chr13b

mafktlqasvkvllllsvcshfisvkmtaaTFIREKCECVKEAGAVQWRKITDYTITPKNPLCNKVQIKLQLSNKEVCLNPESKQGKKLQKCWQKINFNPQRKKVCLTIKKNAPKRLKKL

>z-**cxcl12a**

mdlkvivvvalmavaihapisnaKPISLVERCWCRSTVNTVPQRSIRELKFLHTPNCPFQVIAKLKNNKEVCINPETKWLQQYLKNAINKMKKAQQQQV

>zCXCL-chr13c

mrcsvfvflacmtllsttevfaARLPIQQLRCQCVKTYKGKPINPKLIQSLQTIPAGARCKNMEVIATVKNGKTCLNPKDEWVTKIIEGRSVKAPTRGPIITLPPNSTSVPQLTSKM

>zCXCL-chr13d

mnvllmlsvvfgvsitlvagAVQPLGAGYNSRCVCLKLESRVIPQDNLRRVVILPRGPHCKTTEVIAGLTSGERICLNPRTHWVKKLIMFIEKKKQENNKL

>zCXL-chr12a

mnvllmrlssashqmivssglllllcvftsgvfipesDRIQDTSSPNKNVDARPSLCFQVLTTVEPRKNITS**C**YNLSKKGNCLQ**C**VLFVDAENRMMCMDPNASWLPARLNRLKAKGVT**C**KEWS

>zCCL-chr11a

metqsstmkfqilalllllacmypsiaqgYYENCCLKYVTGIKKNMRRNIMSYRVQLTDGGCNIPAVVFKMRLKRQLKPKSVCADPRSDWVQAIIKELDEKNKRAM

>zCCL-chr11b

mflqtvsilflsavlfgclegKGVQMQRDVQCCMQYSHGKVRTKDVLRYERQTEGPDCSIRAIILYTKKAVKCADPRDRKVKRLLRKLNQRLGAKARRTMWLHPHLNLPVMSEVAVVNSQKMNKTK

>zCCL-chr10a

mmlsnsivaaallilsvslwscttalgDDAVDCCLTTSDRRIPQKVVTTFTLQTGEGGCRVPATIFVTKKGLKLCAPFPSQNNWVSRLIDHILGREKPAQKRPRKSKGKKQRQQ

>zCCL-chr10b

mdlivlgillciafssaqgVTPRCCVETTKRFPLDLLKKVNRYEVQTSSGACTIDALVLHVGDMRYCATPKMEQFLQKLMKRMARLKASAV

>z-**ccl1**

mefrsscllllvcftiiiltdnkgaaIPTCCLSVLRRIPKRVLRSVRTYEVQDTSGHCEIKALILHFKGKKICAHPKLERFLKKMLKHKPKKP

>zCCL-chr7a

mghycvyllvgllaitflqaDVMGNHANTPDACCFTFFKRKIHPSKINSYNPTRVDCTLPGVIFVTQKGLRLCVEPKLNWVKKTIQIIDDRNI

>zCCL-chr5a

massimsafclavsalllcfyssptvaQADLALDCCLTVSPRVIPKHVILAYQKQSRGDGCPRDAVIFITRKGLNLCAPPASEESWVRDTMTFLDKRREKCKETRFIERRCHALKFMKF

>zCCL-chr5b

mllrrtntkttmqtstitllliaavfcinteaFPDTAVDCCLTTKDTRIPLQIVASYFHQTTESGCPIAATVFITKKDKKLCAPPEKNTWISRIISHLEKKQRKALQ

>zCXCL-chr5a

malrpslllavtavccftiiiecLYDCFSALPMDGFATENKCKCQTTTSSRIPPRLFQKIEILPAGAHCRKAEIIITKKDNQAVCLHPEARWVKEMVSKIISKRAERETAMPTVA

>zCXCL-chr5b

mktaaafvalgcflmvevkgKIPDLKNRCLCADKGANNVNLKTIEKIQIIHPSPSCKRLEIVVTLMKGAGKKCLNPESNLGKNILKALRKKKLTAVRRMNPA

>zCXCL-chr5c

mkvsaclinqrllkkgnifpelpvrgsmnqivlillcallfgmslaQSVGHGGGGSQRCRCIGKPYKTVNPRSIQAVDVFQPSPSCSNKEIILTVVEGRGKTKGKGSRKRSKVCLDPNGKQGQRLLKGRWGKKQNQRNRGKKEKNKV

>zCXCL-chr5d

mktvtalllvslavvaiegQHMKSQRCVCLGAGLNMVKPVLIEKIEILPSSPSCGHMEVIATLKNGAGKRCLNPKSKFTKKIIDKIEKNNRNAR

>zCXCL-chr5e

mktlaafllltcliagkvngQDNTSRARCFCADKGINMVLLKNIEKVEIFPPSPSCNKNEIVVTLKNGAGQKCLNPDSKFTQNVVLKAIGKRMQQSVPHSTTTGTVKSSMTSSTSAPTAFK

>zCXCL-chr5f

mktlaavvllgyllvikvegQARAPRSRCLCADKGVNMVSPKLIEKVDIIPPTPSCGNLEIVVTLKNGAEPKCLSPDSKFTQKYLMKALEKRTLQK

>zCXCL-chr5g

mktaaflvflacllatqvhgQKKFNRCSCVGKGLDRVALRNIEKFEIIHPSPSCGKQEIIVTMKSSEQKCLNPESKFTQELIRRALEKRVILTDEVSVVFSGSTSR

>zCXCL-chr5h

mktlaaflllscliagevngQDRSSRARCFCVDKGLNMVLLKNLDKVEIFPPSPSCNKHEIVVTLKNGAGQKCLNPDSKFTKNVVLKAIGKR

>zCXCL-chr5i

mktlaaflllscliagevngQDRSSRARCFCVDKGLNMVLLKNLEKVEIFPPSPSCNKHEIVVTLKNGAGQKCLNPDSKFTKNVVLKAIGKR

>zCCL-chr2a

mnftlftavflcigwivevagNGRPANCCSLKDTKIPAENIVDFNIQEAPPCHIKAIRFYTRKNKVICSDPNSHWAKKMIEKLSLTKDTPKTPIQCHVTKAIQTTTSKTPGTQTETSTSTTVQPRVTVINTSGPETSTSTTTATSEAETSTSSTITTSRPETSRSTTAMTSKKSTKKSEPPPQTYLNTMTTCGTGELMTPAKEKKLSGNIVTSKTPAKRTKPSRLKLKARNKSKKEFRKLQMKKKPK

>zCCL-chr2b

mkfnqfaaflfsiqwvifgNEQQVYGNTSPPGCCLTVKNIRIPAFNIVGYSIQEIPLCSIKAVRFYTKKNRVICSDPNSDWAKKVIQQLSPTPALKKALQCYTNSIQTTTATHKIPGTHSDTSTAVQPTVTIINTFEPETSSNINTFGPETSSKINTFEPETSSKINTFGPETNTNNYFCLKKEKKAIHFCIKQA

>zCCL-chr2c

mtrqiftacvlvvilgsisvfaDGPPMSCCLRLRDRKLHLDKILNYRIQTEDLCPIRAVLFQTVAGKTLCSDPESSWTKSAMWKVDEEQRKLRGQIPEAVEGASVDGCKGREDPMTTAEMPLNSRVLLTKMQTTKQKNSQTTAKAQKEVK

>zXCL-chr2a

mklhvsgfsavlllwllvsssvqEDAHKTGCLSTTDTKTPHTNLRSYTIQQKPLFPVHAVRFLTLKGITICSDPTSPWAIKAMKHLNGKKKQRQSNITIRPSVKVVHMDTSTTNMARVSAQLKKQT

>zCCL-chr2d

mqlnqkmmmrlaaiavimsvmimktngQNRFVQCCTSVSTKEITLPITGFKYQKRNPPCVKAVIFFTKEGEQCIHWNQSWVREKIQELIISMEKMNSTVSTPLKMNSTLSTLLRINSTISTALQMNSTLSTPLSTSSS

>zCCL-chr2e

mphnqkmmmrlaaiavilsvmimetngQNRFVQCCTSVSTQKIILPITGFKLQIRNPPCVKAVIFFTTEGPRCSHWKEGWVKEKIQELKTFQVWEEKMNSTDSIPLSTTTL

>zCCL-chr2f

mslvsitlisivflsllphtpkaYGPLNYACCVKYTRTPLPFGVIAGFIEQSSLEVCRIDAIIFITQKNKKICASIEDQWVRAALARLR

>zCXCL-chr1a

mtskiisvcvivflafltiiegMSLRGLAVDPRCRCIETESRRIGKHIKSVELFPPSPHCKDLEIIATLMTTGQEICLDPSAPWVKKIIDRIIVNRKP

>zCXCL-chr1b

mtskiisvcvivflafltiiegMSLRGLAVDPRCRCIETESRRIGKHIKSVELFPPSPHCKDLEIIATLMTTGQEICLDPSAPWVKKIIDRIIVNRKP

>zCXCL-chr1c

maftpkallllllavvyvqqgevlaKIPDRCQCEESSLVNRARRDTIKEFYITPKRPNCDKVEIILTQKPENKTTASGQLCLNPQKQQGQLLQNCWTRLNINNTDSLKMSVCWQ

>zCCL-chr1a

mnfslftavflcigwivevagNERPKDCCLTVKNIRIPAENIVGYSIQDTPLCSITAVRFYTIKNKVICSDPNSDWAKTVIGKFSPSTKPPQCHTKTIQTTTHKTPGTQTEKSTSTIVQLRVSVSSTSRPHTSTTTTISEAETSTSTTVTTSRPKTSRSTTVTTIQPSQPKTKKSTRRIKPSETPSQTSLNTIICETGEPMTPTSEKTTPGYRVTSTTPARRTNPSRLKLKARKKSKKDFRKLQMGIKPKNKSE

>zCCL-chr1b

mtgriftacvlvvifrsismfaDGPPVSCCLRHGDRRPHLDKILNYRIQTEELCPIRAVLFQTVAGKTLCSKPESSWTKSAMWKVDEEQRKLREQDPEALEGASVDERKGEEHNSRNTTKQQSYNNNADD

>zCCL-chr1c

mnfslftavflcigwiveisgNYPPAHCCPEVTNVRISVEKIVSYSIQEPPLCPIKAVRFYSKKNNVICSDPNSDWAKRVIYNLSPTTVTHKVIQCHTTKTTQTTTQKTPGTKIETSTRTATVQPTVTVINTSGPETSTNTTVTTAETYRPEYSRTTILHTSGLEMDDRKTTETTETHQQDPLNMVEEKQVTLATSVTLLAEVDDANKSTNYFCLKKEKA

>zCCL-chrUa

mmsicwklalmmmtvlvtsQKASVFTNAIEEKGNNCCKSVSTVVVTDPIIGVRIQPESIPCVKAIIFETDRGHFCSDSRQPWARRKVLQFFRTLKKTFN

>zCCL-chrUb

mmsicwklalmmmtvlvtsQKASVFTNAIEEWGNNCCKSVSTVVVTDPIIGVRMQSESIPCVKAIIFETDRGHFCSDPRQPWVRRKVRQFFRTLKKRHSTDIFTTIIH

>zCCL-chrUc

mkiimktallfavlccsllpqssdgQESIDAVNSICCFGEGSNSRIPPKRVEYYYWTSSRCPLKHVVFVTQAKGHLCMNPDNKWVQKVIGMKSGPGSSV

>zCCL-chrUd

mrslmfllvlvlfcslqdtssaTDAISLANSVCCEGITHKKIPLKQIVSHHLTTSNCAIKAIVFTTKAGKSFCVDPGNTFVKRQVAELDSRTRV

>zCCL-chrUe

mssppcpwmsttttlapattaITTRAKTTTLPKPKRQIDRSSIPGPAAVPITCCFAFIDFPIPYNKIVSALRTSPRCATKAIVVTTPRTQFCVKPNEVWIKAVMKKQLQK

>zCCL-chrUf

MGVSRMQEDGPPVSCCRLGDIRPHLDKILNYTIQTEEMCSIRAVLFQTVTGKTLCSDPESSWTKSAMWKVDEEQRKLREQDTVAAEGASVDGRKGREDPMTTAEMPINSRVTTTTMKSKEQPPDCCLTVTNIRMPLEQIVAYSL

>zCCL-chrUg

mkfnqfaaflfyklwmisgneqqvygNERPPDCCLTVKNIRIPAENILAYSIQEAPSCGIKAVKFYTKKNRIICSDPNSDWAKTVIQKLCLTPAHKNIECHANSVQTTTATHKIPGFTLLPTENGPDSYRQ

>zCCL-chrUh

mktpllllvcvvmlcslpdsssgQESIDAGNRICCYGKGSNIKILLKRLESFYWTSSNCPLSHIVFVTISKRHLCMNPDNEWVKKVINMKPGSKN

>zCCL-chrUi

mghccvyllvgllavtflqisgigNNGNNPTECCINYFQRRIPFDRIECYIETRIECRNPGVIFVTKKGLRLCVDPQLKWVNKTIYRIDNGSF

>zCCL-chrUj

mripvfllflvftmcsiqlvpaYPVKETCCFNFIDFPIPANKIMFVARTSSRCAVKGIVVSTPRTQFCVKPDEDWIKPIMEKQYKR

>tCCL-chr18a

mrtapalllcllaagllsfascRNEIGPDDCCFRFYPHRVKRTLVRSYYATDQRCSKTAVILVTQRSRHICVDPNLSWVETLLKNLEESSF

>fCCL-chr15a

mrvsalffltvtacicltlaQVTYDDCCMKYAKKMRPKMQKHVINYRWQVPDGGCNLLAVIFTTSKGRIRCSDPKEKWVTDLMRDVDQKKLKRRSKTVRHVY

>tCCL-chr15a

mrlnalffltitacvclalaQITYEDCCLKYIKKVKPRIQKYAVSYRLQVLDGGCNLPAVIFVMKKGRVVCTDPKEQWVTELMRQIDGRRLRTHSNKSTKHNSRG

>fCCL-chr15b

mitvttfvlcfvlaltpapyalgSHASRSCCTRYSGKPVPPQLIRGYREHTAMENCRIEAIIFYTVQRKMVCANPKDEWVKKVLKLLRWQNLPVKNAPPALKAPIETARNCRWQKGRLKHVPATAKLPRRQ

>tCCL-chr15b

mitvttvvlcfflvltpahyAPGSHASSGCCTRYSRRPVPFQLIKGYREQTTMENCHIEAIIFYTVQRKMICASRKDEWVRKLLKLLRWRCRLVKNGPAGLKRHR

>fCCL-chr15c

mgklmlcvsvlvlllaltesSYFCCTQYHENPVSVEILKFYMIQEDTGYCNIRAVIFKTKINRKPLCANPESSWVKDAMETVPQ

>tCCL-chr15c

mmklmlglsvlvlllaltesRYFCCTQYHEKPVPVKMLKYYIIQEDTGYCNIKAVIFKTKTKPLCANPESWWVKIAMETVPL

>fCCL-chr13a

mktvclvvalllvticcvsaMPKAMDPIASPTKCCLGFSEGRLPAQNVRDIYKTHQGCRKKAFVIKMKRGEFCYDQRNEWALNLYSQFHSPPTSHH

>tCCL-chr13a

mktlclavtlllltvcccnaMPSALPLPENVRCCAQFTEQPVPKRNVRKIYKTSHQCGQKAFIVETLRRELCYRQSFPWALKVYKEFSDTADIQ

>fCCL-chr12a

masraaallllalvciefaaaEVVLDCCKTKTSKFFPLQRIQSYRIQDSGTGCDIDATVFVTKNGRHLCVSHPSEEKWVKKHIDALEKRKQK

>tCCL-chr12a

masraaallllglvcvqfaaaQVVLDCCRTKTSKLLPLQLIRSYSVQDAGAGCDISATVFVTKTGRQLCVSHPSEEKWVQKHIDALLRRKEKHAKTRVE

>fCCL-chr12b

masraaallllalvciefaaaEVVLDCCKTKTSKFFPLQRIQSYRIQDSGTGCDIDATVFVTKNGRHLCVSHPSEEKWVKKHIDALEKRKQKVE

>tCCL-chr12b

masraaallllglvcvqfaaaQVVLDCCRTKTSKLLPLQLIRSYSVQDAGAGCDISATVFVTKTGRQLCVSHPSEEKWVQKHIDALLRRKEKHAKTRVE

>fCCL-chr12c

mapsgdakllfciffiscccltvtlaEVPVDCCLSVGKQQVIKHAIVDYHRQVAGQGCSLNATILVTRRQVRLCVPANEQWVEKVVEHVEKLRAHCRKNKYKKPLCSKMMSRN

>tCCL-chr12c

malfgdaklvlclffaiccymtvtqaEVPADCCLSVTNAEVIKHAIVDYRRQVAGQGCTLNATILVTRRQKQLCAPASERWVEDVVAHVKQLRKCCNKAKCRQANKKKRCLGVKAE

>fCL1

mdlkvvavliclsafaisstqaAIPGCCINTRKIIPINVLRKVSRWTIQSSGGACDIDAVILHVRDKRICVDQTVFKDIWWRMKQWKQRVKKRAAKYNV

>tCL1

mdlkvvavlfllsafaissseaGIPRCCTSTRKGIHPKDVMKVVGWNLQRSGACDIDAVVLLLKNKRRMCLDWGVFKAVFPAEEKRLMKKRTAK

>fCCL-chr1a

mklqtlfllllftcmyvslaqgQSYGNCCLGYVNSMRRNAKKNIERYWRQETDGDCNIRAVVFQMKKKRSQQKQRTVCTNPEHDWVQELIAAVDARVAKQN

>tCCL-chr1a

mklqtlfvlllftcmymslaQGSYGNCCLGYVPAMRRNAKNIERYWRQETDGDCNIRAVVFVMKKKQGQKKPRTVCANPEQTWVQSLMAHVDGREGKIN

>tCCL-chrUa

mmktlrlavalllltayccngMPSALPPPSTVKCCLKFAEYAVPLGNIKNVYKTSDFCKEDAFIVETPRRELCYRQSFPWALKVYKEFSDTADIQ

>fCCL-chrUb

maitlitvcvlathtslaHPGCCSSYMKSRIPFRIIKGYSVQTVTEICPIDAIIFGKSLYQSCFEMGDGIHSKIKVSFTLVLIIFFCIFYSV

>fCCL-chrUc

mvmlqmltvisltvillasvegKGVQMQKDVQCCMLYSQGKVRTKDVLRFEVQTEGPDCSIQAIILYTKKAVKCADPRDRKVKRLLRKLLQRQRTKAHRTMWLQPYDNLPVMTEVRENL

>f-**il8**

mcsrvfltslvvllaflaisngMSLRSLGVEQHCRCIQTESRPIGRHIGKVELIPPNSHCEETEIIATLKMSGQEVCLDPKAPWVKKVINKIMSSRQR

>t-**il8**

mcsrifltslvvllaflaisngMSLRSLGVEQHCRCIETESRRIGRHIGKVELILPNSHCERMEIIATLKKTGEEVCLDPEAAWVKKVIERFLSRMALTICTRFTDEYQLFEELGK

>fCXCL-chr17a

melhlptvrqltflslccvlatvtqsDSTFVPGRCLCPRTQPAVRGPLKNLTVYYTNPTCDRVTVIVTLRSNDTEVCLDPEAPMGRRLIHCWKRAHKVGRDVKNCLRRRRNARPGRRPGQGSPQRTRGQGRKSLS

>tCXCL-chr17a

melhlpsarqlvllslgcvlitvrqsdgTFVPGRCLCPQTQATVRGPLRALSIYHRNPTCNKVTVIVTLRNNDEVCLDPKAPLGKRLIHCWRRTQKKGRDVRHCLRRRRRNVKQGGRPGQGSPQSSRGQGRKSSSS

>fCXCL-chr17b

mdvklmtlvaaimvtlnappsqaKPISLVERCYCRSTVSNLPRSYIRELRFIHTPNCPFQVIAKLKTNKEVCVNPEIRWLQQYLKNAINKMKKLKHGN

>tCXCL-chr17b

mdvklvtlvaalmvvlyappsqaKPISLVERCYCRSTVSNLPRAYIRELRFIHTPNCPFQVIAKLKSNKEVCVNPQIQWLQQYLKNAINKMKKFKQGN

>tCXCL-chr12a

makhvtlllvlmlcchqapadaFSGCHCLRIFRRPIPFRIIKQVEMIPISGQCRRPETILTRRNGSKDCIDPNQQWFKDVLRKITVPNSRNVTKNATKPGNF

>fCXCL-chrUa

mklcillvfatmlavitgMPPISRDYNNRCRCLQVESRIIPPDNLKSIKLITEGPHCPEKEVIANLVNGAKVCLNPKSTWVKKLVHFVLGKQLSRKQATLPKA

>tCXCL-chrUa

mkicilfvfatllaastgMAPISRDYVTRCQCLQVESRIIPPDSLRSIKLIPEGPHCPTAEVIAGLASGAKVCLNPKSTWVKKLVQFVLEKQLNRKQATLPKTKA

>fCXCL-chrUb

mhrctavllllavslyflgaeaYKCRCTRKGPKIRYKDVQKLEIKPKYPYCQEKMIFVTMENVARFKGQEYCLHPKLQSTKNLVKWFRIWKDKHRVYEA

>tCXCL-chrUb

mhrctalllllvvslyvlgaeaYKCRCTRKGPKIRYKDVQKLEIKPKHPYCQEKMIFVTMENVARFKGQEYCLHPKLQSTKNLVKWVYEA

>fCXCL-chrUc

mvkpptllvvmtlccclitadaFFGCHCLRTIRKPIPLNVIEKIEMLPISGHCRRPEIILTRKNGSKICIDPNQKWFKDLLNKM

>tCXCL-chrUc

maklltlllvlmlcchqapaDAFSGCHCLRIFRRPIPFRIIKQVEMIPISGQCRRPETILTRRNGSKDCIDPNQQWFKDVLRKITVPNSRNVTKNATKPGNF

>fCXCL-chrUd

mmrttvalciflaciavctsSPACRCLNTVAAVNPSHVVDVVEYGPRPYCRRQEVIVILKNKRPRCLDPKGQFAQGLLWAKR
