# Supplementary figures and images for: Extensive expansion and diversification of the chemokine gene family in zebrafish: Identification of a novel chemokine subfamily CX
Source: BMC Genomics. 2008 May 15;9:222. doi: 10.1186/1471-2164-9-222 (PMC2416438; doi:10.1186/1471-2164-9-222)

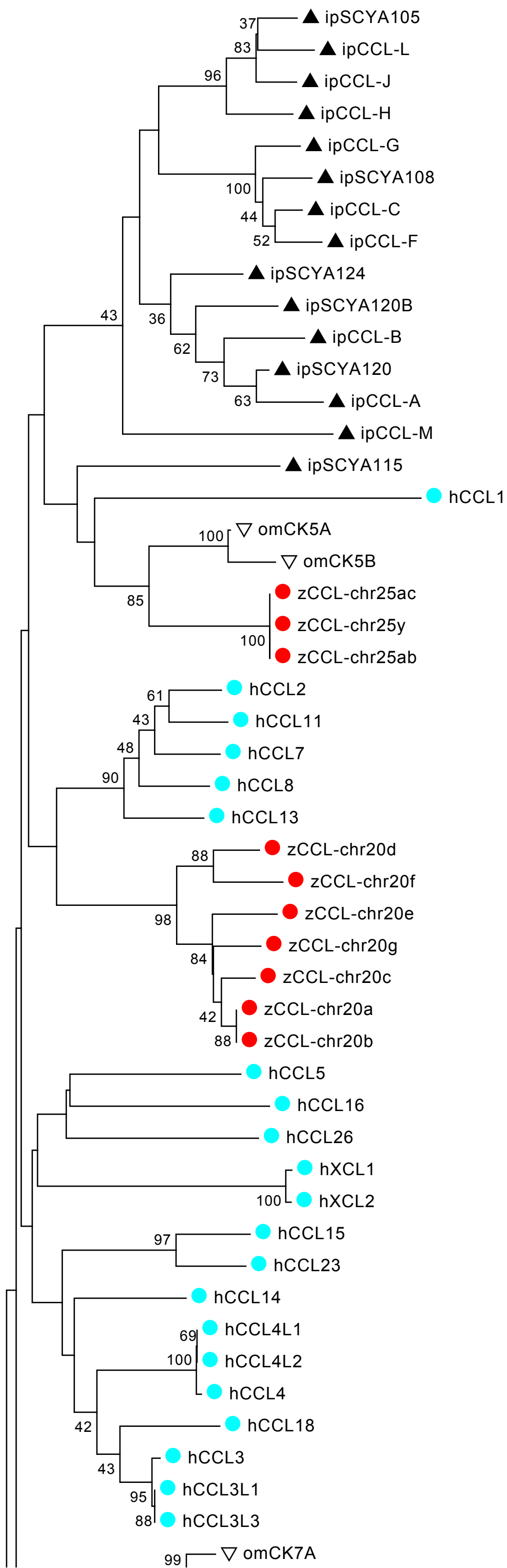

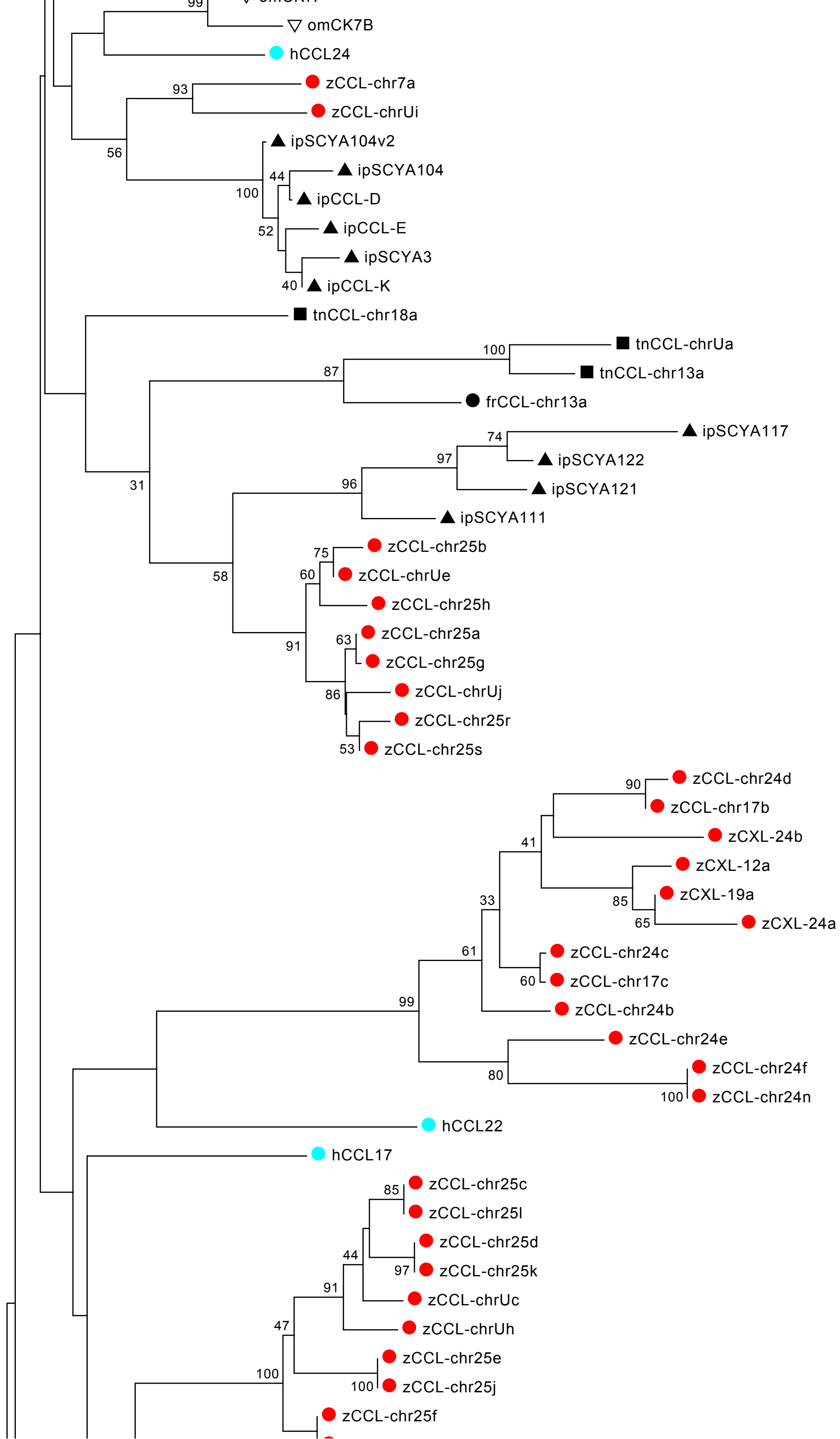

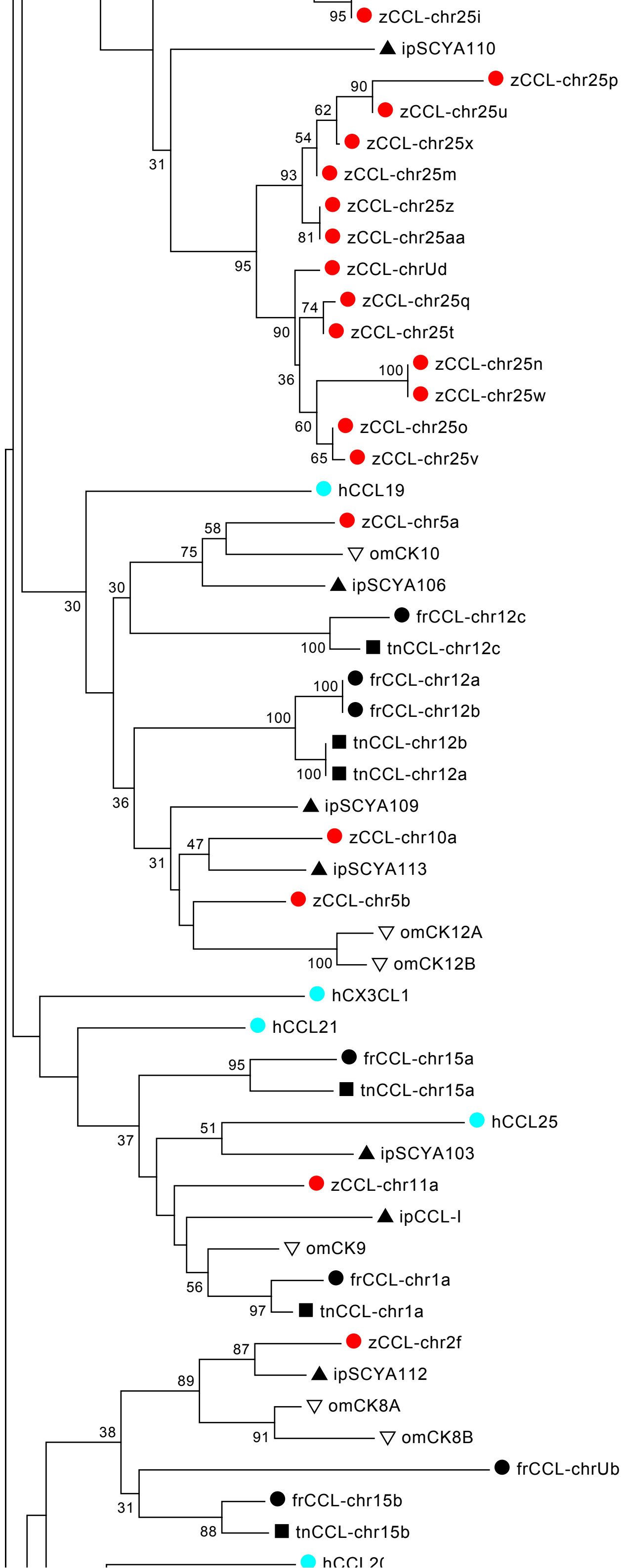

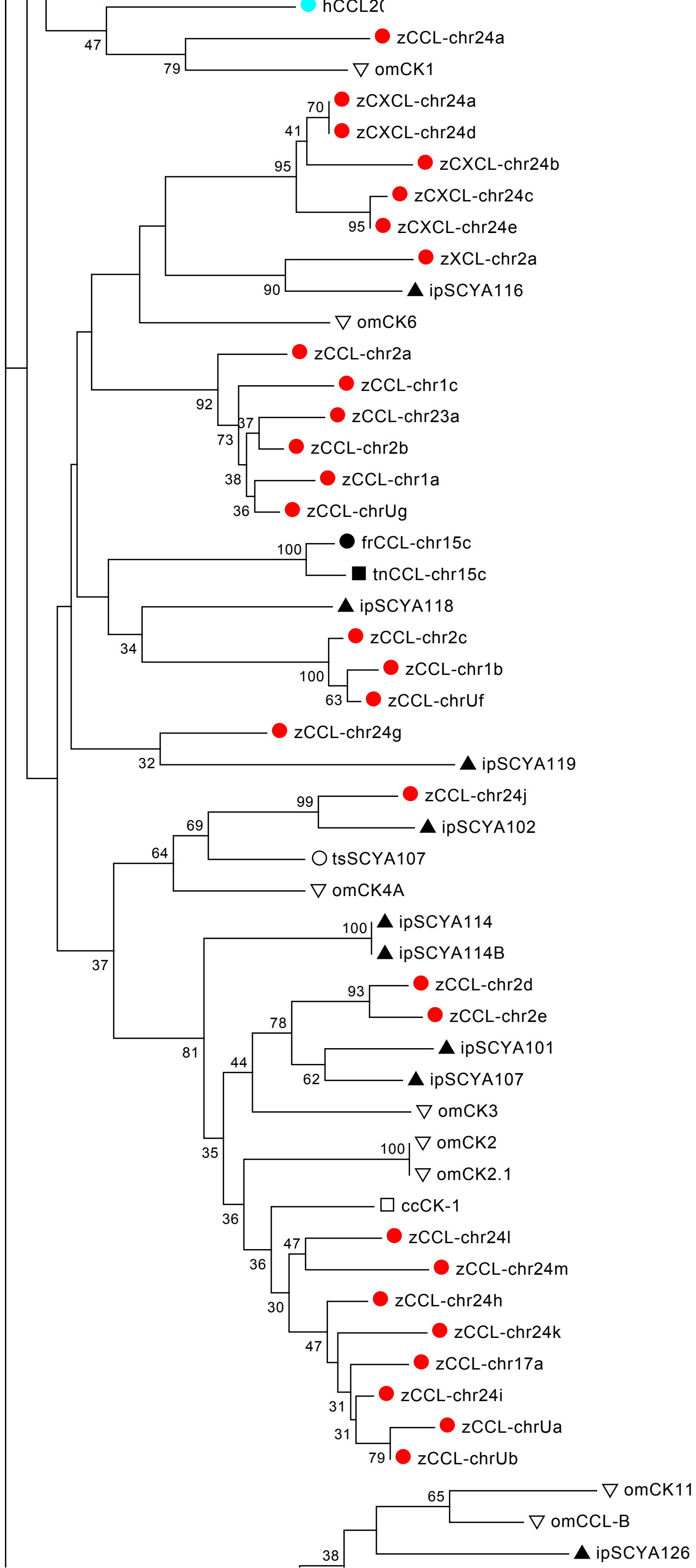

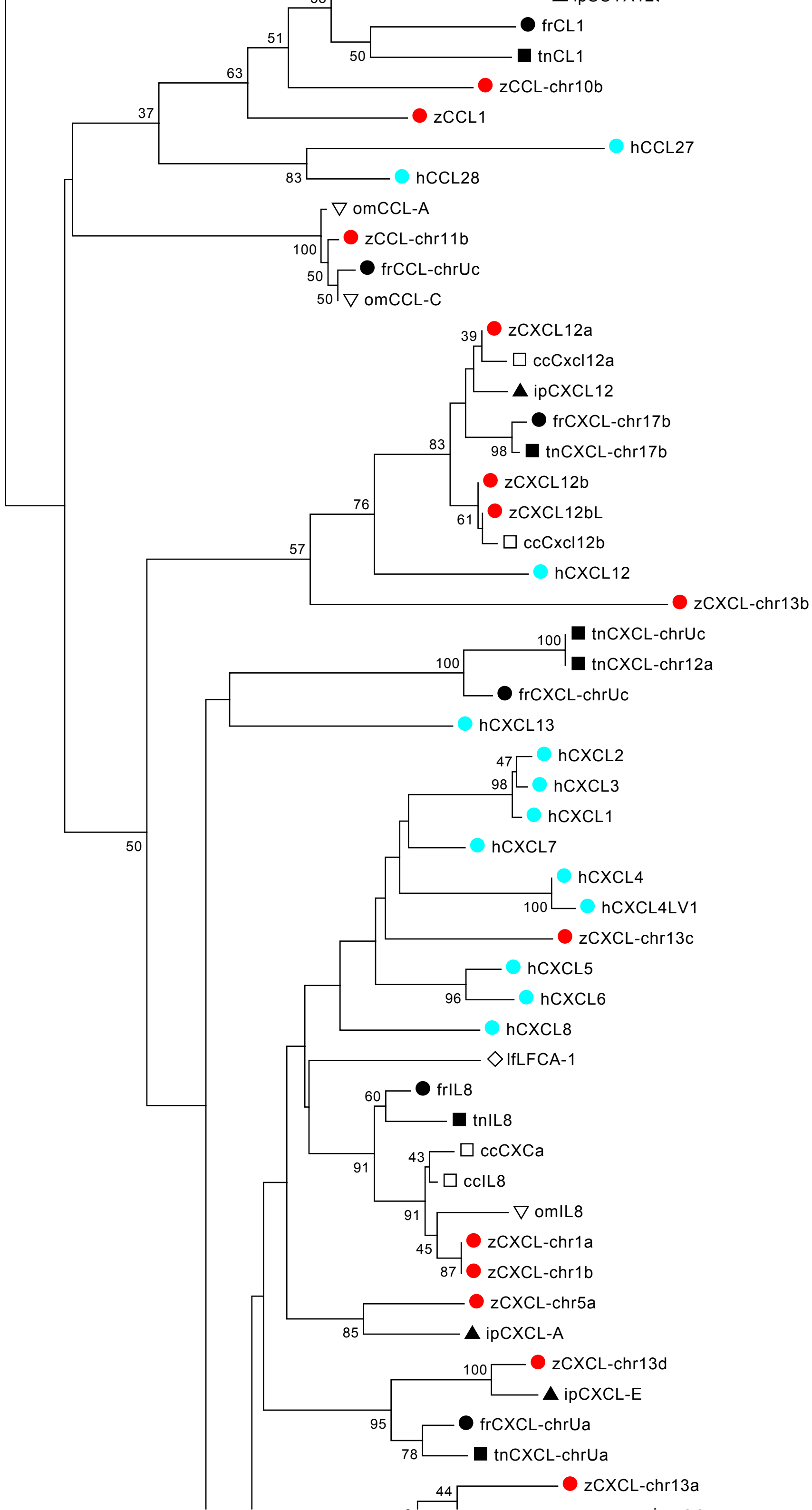

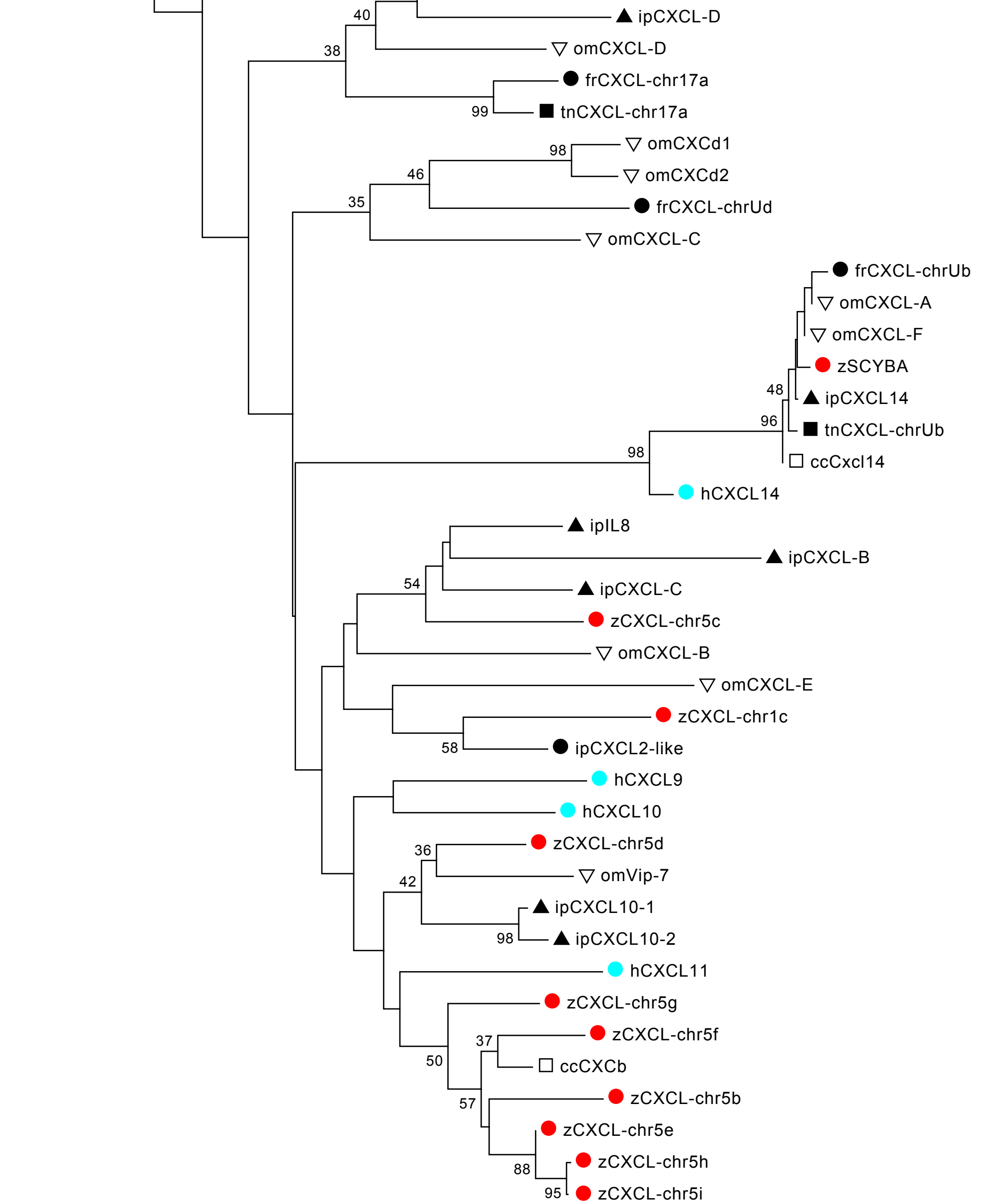

0.2

Supplement: Additional file 4 — A phylogenetic tree constructed using all chemokine subfamily members. The tree was constructed using the Dayhoff matrix and the neighbor-joining method. Numbers at branch nodes represent the confidence of bootstrap test with 1000 iterations. Confidence values of ≥30% are shown. The symbols used are the same with those in Figure 2. [file 1471-2164-9-222-S4.pdf]
